# Spline Analysis of Biomarker Data Pooled from Multiple Matched/Nested Case–Control Studies

**DOI:** 10.3390/cancers14112783

**Published:** 2022-06-03

**Authors:** Yujie Wu, Mitchell Gail, Stephanie Smith-Warner, Regina Ziegler, Molin Wang

**Affiliations:** 1 Department of Biostatistics, Harvard T. H. Chan School of Public Health, Boston, MA 02215, USA; yujiewu@g.harvard.edu; 2 Division of Cancer Epidemiology and Genetics, National Cancer Institute, Bethesda, MD 20814, USA; gailm@exchange.nih.gov (M.G.); zieglerr@exchange.nih.gov (R.Z.); 3 Department of Epidemiology, Harvard T. H. Chan School of Public Health, Boston, MA 02215, USA; swarner@hsph.harvard.edu; 4 Department of Nutrition, Harvard T. H. Chan School of Public Health, Boston, MA 02215, USA; 5 Channing Division of Network Medicine, Brigham and Women’s Hospital, Boston, MA 02215, USA

**Keywords:** between-study variation, calibration, dose–response curve, nested case–control study, pooling biomarker data, pooling project

## Abstract

**Simple Summary:**

This paper presents methods to pool continuous biomarker measurements from multiple studies to estimate the dose–response curves that allow for the nonlinear association between biomarker values and disease risks in matched/nested case–control studies. The approach can be easily applied to pooling projects of cancer studies and user-friendly software for implementing the method can be found on the corresponding author’s website.

**Abstract:**

Pooling biomarker data across multiple studies enables researchers to obtain precise estimates of the association between biomarker measurements and disease risks due to increased sample sizes. However, biomarker measurements often vary significantly across different assays and laboratories; therefore, calibration of the local laboratory measurements to a reference laboratory is necessary before pooling data. We propose two methods for estimating the dose–response curves that allow for a nonlinear association between the continuous biomarker measurements and log relative risk in pooling projects of matched/nested case–control studies. Our methods are based on full calibration and internalized calibration methods. The full calibration method uses calibrated biomarker measurements for all subjects, even for people with reference laboratory measurements, while the internalized calibration method uses the reference laboratory measurements when available and otherwise uses the calibrated biomarker measurements. We conducted simulation studies to compare these methods, as well as a naive method, where data are pooled without calibration. Our simulation and theoretical results suggest that, in estimating the dose–response curves for biomarker-disease relationships, the internalized and full calibration methods perform substantially better than the naive method, and the full calibration approach is the preferred method for calibrating biomarker measurements. We apply our methods in a pooling project of nested case–control studies to estimate the association of circulating Vitamin D levels with risk of colorectal cancer.

## 1. Introduction

Pooling biomarker data from multiple studies can result in more precise estimates of the associations between the biomarker levels and disease risks due to increased sample sizes and can also facilitate subgroup analysis [1,2,3]. Examples of pooling project examining biomarker-disease associations include the Circulating 25-Hydroxyvitamin D (25(OH)D) and Colorectal Cancer [4], the 25(OH)D and the Risk of Rarer Cancers [5], the Endogenous Hormones, Nutritional Biomarkers and Prostate Cancer Collaborative Group Studies [6,7] and the NCI Breast and Prostate Cancer Cohort Consortium [8].

A statistical challenge in the pooling analysis is potential between-study variation in the biomarker measurements stemmed from assay or laboratory differences. For instance, the measurements of serum 25(OH)D concentration can vary from 20–40% across different assays, laboratories and even seasons of a year [9,10,11]. Also, hormones such as estradiol and testosterone have high variation across assays and laboratories [1,2,3]. Sloan et al. [12] proposed methods for pooling biomarker data from matched/nested case–control studies based on the regression calibration approach [13,14], which is widely used for handling the covariate measurement error problems. To be specific, a calibration procedure is performed by first selecting a reference laboratory, to which a subset of biospecimens from each study are sent for re-assaying. A calibration model is then fitted for each study based on the study-specific local laboratory and the reference laboratory measurements among the subset of biospecimens selected for re-assaying. The models are used to impute the reference laboratory measurements for the remaining observations with only local laboratory measurements available. Due to their rarity, cases are not usually used for assay calibration. Instead, controls are usually chosen for re-assaying in a reference laboratory [15].

However, the existing methods assume that biomarker measurements have a linear relationship with the log relative risk (RR) of diseases [12]. The nonlinear dose–response relationship between biomarker measurement and disease risk is often observed in practice [16,17]. Restricting the biomarker–disease relationship to be linear may lead to biased estimates when the true association is nonlinear. In this paper, we proposed the method for pooling matched/nested case–control studies to allow for a nonlinear relationship between biomarker levels and log relative risk of diseases by using spline functions [12,18]. We first calibrate biomarker measurements across multiple studies. We then obtain estimates for the coefficients of the spline functions based on an approximate conditional likelihood. We also propose an analytic variance formula for the estimated parameters of interest, which takes account of the uncertainty due to estimating the calibration parameters.

In Section 2, we present the models and statistical methods. In Section 3, we evaluate the performance of our methods in simulation studies. In Section 4, we apply the methods to a pooling project investigating the relationship between circulating Vitamin D levels and colorectal cancer, and finally, we present a discussion in Section 5.

## 2. Methods

### 2.1. Notation and Model

The logistic regression model with spline functions can be written as:

$$\mathrm{logit}\left(P\left(Y_{sji}=1|X_{sji},Z_{sji}\right)\right)=\beta_{0sj}+\beta_{X}^{T}f\left(X_{sji}\right)+\beta_{Z}^{T}Z_{sji}\;\;,$$

where s∈{1,…,S} index the studies in the pooling projects, where the first *Q* studies only use local laboratories for the measurement of biomarkers and thus require calibration; $n_{sj}$ and $m_{sj}$ denote the number of cases and controls, respectively, in the *j*-th stratum of the *s*-th study, and within each stratum, $i\in \{1,2,\ldots ,m_{sj}\}$ denotes controls and $i\in \{m_{sj}+1,m_{sj}+2,\ldots ,m_{sj}+n_{sj}\}$ denotes cases. $Y_{sji}$ denotes the binary disease status; $X_{sji}$ is the biomarker measurements from the reference laboratory, which is included in the model through a nonlinear function f(·), and $Z_{sji}$ is a *p*-dimensional vector of potential confounders. $\beta_{0sj}$ is the study and stratum specific intercept, and $\beta_{X}$ and $\beta_{Z}$ are vectors of the corresponding regression coefficients. Without further specification, all vectors are column vectors in this paper.

Note that, in nested case–control studies with density sampling [19], $\beta_{X}^{T}\left[f\left(X^{*}\right)-f\left(X^{**}\right)\right]$ represents the log relative risk (RR) comparing two distinct biomarker levels $X^{*}$ and $X^{**}$. We focus on methods for point and interval estimates of $\beta_{X}$ in this paper. To model the possible nonlinear relationship between the biomarker and disease risk, we propose to use the restricted cubic spline method [18]. Our approach works for other spline functions as well. The restricted cubic spline method has advantages of being parsimonious, while allowing for flexibility in characterizing nonlinear curves. Typically, the model fit is not heavily affected by the location of knots, but depends more on the number of knots selected. The recommended numbers of knots are 3, 4, 5, 6, and 7 [20].

Let $W_{sji}$ be the biomarker measurements from local laboratories. For each study using a local laboratory, a subset of samples were selected and sent to the reference laboratory for re-assaying to obtain reference biomarker measurements; we refer to this subset as the calibration subset. Therefore, for studies that use local laboratories for biomarker measurements, $X_{sji}$ are available only in the calibration subsets, and $W_{sji}$ are available for all individuals. Since the local laboratory measurements can vary systematically across different studies, using $W_{sji}$ instead of $X_{sji}$ in data analysis can lead to biased estimates of the biomarker–disease relationship.

### 2.2. Approximate Conditional Likelihood and Calibration Methods

Let vectors $X_{sj}$ and $W_{sj}$ and matrix $Z_{sj}$ contain the corresponding measurements of individuals from the *j*-th stratum of the *s*-th study. The conditional likelihood contribution from the *j*-th stratum of the *s*-th study using the reference laboratory measurements is:

(1) $$\begin{array}{cc} L_{sj}^{*} & =P(Y_{sj1}=0,\ldots ,Y_{sj,m_{sj}}=0,Y_{sj,m_{sj}+1}=1,\ldots ,Y_{sj,m_{sj}+n_{sj}}=1|X_{sj},Z_{sj},\sum_{i=1}^{m_{sj}+n_{sj}}Y_{sji}=n_{sj})\\ \; & =\frac{\prod_{l=1}^{n_{sj}}\exp\left(\beta_{0sj}+\beta_{X}^{T}f\left(X_{sj,m_{sj}+l}\right)+\beta_{Z}^{T}Z_{sj,m_{sj}+l}\right)}{\sum_{\left(i_{1},\ldots ,i_{n_{sj}}\right)\in A_{sj}}\prod_{l=1}^{n_{sj}}\exp\left(\beta_{0sj}+\beta_{X}^{T}f\left(X_{sj,i_{l}}\right)+\beta_{Z}^{T}Z_{sj,i_{l}}\right)}\\ \; & ={\left(1+\sum_{\left(i_{1},\ldots ,i_{n_{sj}}\right)\in A_{sj}^{\prime }}\exp\left(\beta_{X}^{T}\sum_{l=1}^{n_{sj}}\left[f\left(X_{sj,i_{l}}\right)-f\left(X_{sj,m_{sj}+l}\right)\right]+\beta_{Z}^{T}\sum_{l=1}^{n_{sj}}\left[Z_{sj,i_{l}}-Z_{sj,m_{sj}+l}\right]\right)\right)}^{-1}, \end{array}$$

where $A_{sj}$ is the set of all subsets of indices of size $n_{sj}$ from $\left\{1,2,\ldots ,m_{sj},m_{sj}+1,\ldots ,m_{sj}+n_{sj}\right\}$; $\left\{i_{1},i_{2},\ldots ,i_{n_{sj}}\right\}$ corresponds to one specific such subset in $A_{sj}$, and $A_{sj}^{\prime }$ is $A_{sj}$ with the subset $\left\{i_{1}=m_{sj}+1,i_{2}=m_{sj}+2,\ldots ,i_{n_{sj}}=m_{sj}+n_{sj}\right\}$ excluded [19].

However, the conditional likelihood function based on $L_{sj}^{*}$ cannot be calculated directly, since $X_{sj}$ is not available to all individuals in the studies that only use local laboratories to measure the biomarkers. To derive an approximate observed conditional likelihood under the matched/nested case–control study design, we make the following ‘surrogacy’ assumption [12] that takes into account of the study design:

$$P\left(Y_{sj}|X_{sj},W_{sj},Z_{sj},\sum_{i=1}^{n_{sj}+m_{sj}}Y_{sji}=n_{sj}\right)=P\left(Y_{sj}|X_{sj},Z_{sj},\sum_{i=1}^{n_{sj}+m_{sj}}Y_{sji}=n_{sj}\right).$$

This assumption implies that the local laboratory measurements $W_{sj}$ do not contain additional information about the outcome, given the reference laboratory measurements, other covariates of interest, and the matched/nested case–control study design scheme.

Under this surrogacy assumption, the observed likelihood contribution from a stratum based on local laboratory biomarker measurements

$$\begin{array}{cc} L_{sj}= & P\left(Y_{sj1}=0,\ldots ,Y_{sj,m_{sj}}=0,Y_{sj,m_{sj}+1}=1,\ldots ,Y_{sj,m_{sj}+n_{sj}}=1|W_{sj},Z_{sj},\sum_{i=1}^{m_{sj}+n_{sj}}Y_{sji}=n_{sj}\right) \end{array}$$

can be written as:

(2) $$L_{sj}=E_{X_{sj}|W_{sj},Z_{sj},\sum_{i=1}^{n_{sj}+m_{sj}}Y_{sji}=n_{sj}}\left(L_{sj}^{*}\right),$$

where $L_{sj}^{*}$ is defined in Equation (1). For Equation (2), we further expand $L_{sj}^{*}$ in Taylor series around ${\tilde{X}}_{sj}=E\left(X_{sj}|W_{sj},Z_{sj},\sum_{i=1}^{m_{sj}+n_{sj}}Y_{sji}=n_{sj}\right)$, yielding the following approximate likelihood contribution from the *j*-th stratum of the *s*-th study:

$${\tilde{L}}_{sj}={\left(1+\sum_{\left(i_{1},\ldots ,i_{n_{sj}}\right)\in A^{\prime }}\exp\left(\beta_{X}^{T}\sum_{l=1}^{n_{sj}}\left[f\left({\tilde{X}}_{sj,i_{l}}\right)-f\left({\tilde{X}}_{sj,m_{sj}+l}\right)\right]+\beta_{Z}^{T}\sum_{l=1}^{n_{sj}}\left[Z_{sj,i_{l}}-Z_{sj,m_{sj}+l}\right]\right)\right)}^{-1}.$$

This approximation performs best when the conditional variance and covariance of $X_{sj}$ are small or when the biomarker effect is not strong. Section S1 of the Supplementary Materials provides a detailed derivation of the approximate observed conditional likelihood and the conditions when the approximation works well.

To obtain an estimate of ${\tilde{X}}_{sji}$, for the studies that use local laboratories, the study-specific calibration models can be fitted in the calibration subsets where subjects were selected for re-assaying in the reference laboratory; these subjects therefore have biomarker measurements from both the local and reference laboratories. The calibration models can thus be used to impute the reference biomarker measurements for the remaining subjects of each study that only have local laboratory measurements.

We make the calibration assumption [12] that, given the local laboratory measurements, the mean reference laboratory measurements are approximately independent from other covariates, that is:

$$E\left(X_{sj}|W_{sj},Z_{sj},\sum_{i=1}^{m_{sj}+n_{sj}}Y_{sji}=n_{sj}\right)\approx E\left(X_{sj}|W_{sj},\sum_{i=1}^{m_{sj}+n_{sj}}Y_{sji}=n_{sj}\right).$$

We further assume a linear relationship between the reference laboratory measurements and local laboratory measurements, leading to the following calibration model:

(3) $$E\left(X_{sji}|W_{sji},\sum_{i=1}^{m_{sj}+n_{sj}}Y_{sji}=n_{sj}\right)=a_{s}+b_{s}W_{sji},$$

where $a_{s}$ and $b_{s}$ are study-specific model parameters. Note that these calibration parameters are the same across different strata in each study. However, we can relax this constraint by including matching factors in the calibration model; that is, assuming $E\left(X_{sji}|W_{sji},M_{sji},\sum_{i=1}^{m_{sj}+n_{sj}}Y_{sji}=n_{sj}\right)=a_{s}+b_{s}W_{sji}+c_{s}^{T}M_{sji}$, where $M_{sji}$ is the vector of matching factors. Sloan et al. [15] suggested that Model (3) is sufficient in most study settings. In (3), although a linear term of $W_{sji}$ is typically sufficient to model the $X_{sji}$-$W_{sji}$ relationship, nonlinear terms in $W_{sji}$ can also be included if appropriate.

The study-specific calibration models are usually fitted only among controls because case biospecimens are often not available in the calibration study subsets. Therefore, the calibration model in practice is typically:

$$E\left(X_{sji}|W_{sji},Y_{sji}=0\right)=a_{s,co}+b_{s,co}W_{sji},$$

where we use $a_{s,co}$ and $b_{s,co}$ to denote the parameters in the calibration model fitted among controls only. Note that ${\hat{a}}_{s,co}$ and ${\hat{b}}_{s,co}$ are generally not consistent estimates of $a_{s}$ and $b_{s}$ in (3). Sloan et al. [12] provide conditions for ${\hat{a}}_{s,co}\approx {\hat{a}}_{s}$ and ${\hat{b}}_{s,co}\approx {\hat{b}}_{s}$ under the bivariate normality of $X_{sj}$ and $W_{sj}$ in a 1:1 matched/nested case–control study. It is straightforward to generalize their results to the $n_{sj}:m_{sj}$ matching scenarios. Specifically, ${\hat{b}}_{s,co}\approx {\hat{b}}_{s}$ when $Var\left(X_{sj}|\sum_{i=1}^{m_{sj}+n_{sj}}Y_{sji}=n_{sj}\right)\approx Var\left(X_{sj}|Y_{sj}=0\right)$, and ${\hat{a}}_{s,co}\approx {\hat{a}}_{s}$ when $Var\left(X_{sj}|\sum_{i=1}^{m_{sj}+n_{sj}}Y_{sji}=n_{sj}\right)\approx Var\left(X_{sj}|Y_{sj}=0\right)$ and $E\left(X_{sj}|\sum_{i=1}^{m_{sj}+n_{sj}}Y_{sji}=n_{sj}\right)\approx E\left(X_{sj}|Y_{sj}=0\right)$. In addition, if the biomarker effect is small (i.e., $\beta_{X}\approx 0$), ${\hat{a}}_{s,co}$ and ${\hat{b}}_{s,co}$ will also be close to ${\hat{a}}_{s}$ and ${\hat{b}}_{s}$.

In studies that used the local laboratory for measurement, for the *internalized calibration method*, the biomarker value in the approximate likelihood ${\tilde{L}}_{sj}$ is ${\tilde{X}}_{sji}=X_{sji}$ if reference laboratory measurement $X_{sji}$ is available, and ${\tilde{X}}_{sji}=\hat{E}\left(X_{sji}|W_{sji},Y_{sji}=0\right)$ otherwise. For the *full calibration method*, ${\tilde{X}}_{sji}=\hat{E}\left(X_{sji}|W_{sji},Y_{sji}=0\right)$ regardless of whether reference laboratory measurement $X_{sji}$ is available or not [12]. Therefore, for studies using local laboratories for biomarker measurement, all participants’ biomarker measurements are calibrated under the full calibration method while, under the internalized calibration method, the biomarker measurements are calibrated for participants who only have local laboratory measurements available.

### 2.3. Parameter Estimation

Define $a=\left[a_{1},a_{2},\ldots ,a_{Q}\right]$, $b=\left[b_{1},b_{2},\ldots ,b_{Q}\right]$ and the dose–response parameters $\beta =\left[\beta_{X},\beta_{Z}\right]$. The collective set of parameters to be estimated is therefore $\theta =\left[a,b,\beta \right]$. The joint estimating equations are $\left[\psi_{a},\psi_{b},\psi_{\beta_{X}},\psi_{\beta_{Z}}\right]=0$, where $\psi_{a},\psi_{b},\psi_{\beta_{X}},\psi_{\beta_{Z}}$ are the estimating functions for their corresponding parameters. Section S2 in the Supplementary Materials contains the technical details.

We can obtain the point estimate of β using a two-step pseudo-maximum likelihood method [21]. In the first step, the estimates of a and b of the calibration models are obtained by fitting linear regressions on the subset of controls chosen for re-assaying in the reference laboratory, and in the second step, β is obtained using pseudo-maximum conditional likelihood method by solving the estimating equations $\left[\psi_{\beta_{X}}\left(\hat{a},\hat{b}\right),\psi_{\beta_{Z}}\left(\hat{a},\hat{b}\right)\right]=0$, where $\hat{a}$ and $\hat{b}$ are the estimates obtained in the first step.

We can use the sandwich variance formula over the joint estimating equations $[\psi_{a},\psi_{b},$$\psi_{\beta_{X}},\psi_{\beta_{Z}}]=0$ to estimate $Var(\hat{\theta })$. See Section S3 of the Supplementary Materials for technical details.

## 3. Simulations

We performed a simulation study for a 1:1 matched case–control study design. Define $\epsilon_{sji}$ as the error term in the linear model of $X_{sji}$ on $W_{sji}$. We assumed a similar multivariate normal distribution of $X_{sji},W_{sji}$ and $\epsilon_{sji}$, as in Sloan et al. [12], such that:

$$\left(\begin{array}{c} X_{sji}\\ W_{sji}\\ \epsilon_{sji} \end{array}\right)∼\mathrm{MVN}\left(\left(\begin{array}{c} \mu_{x}\\ \left(\mu_{x}-a_{s}\right)/b_{s}\\ 0 \end{array}\right),\left(\begin{array}{ccc} \sigma_{x}^{2} & b_{s}\sigma_{ws}^{2} & \sigma_{x}^{2}-b_{s}^{2}\sigma_{ws}^{2}\\ b_{s}\sigma_{ws}^{2} & \sigma_{ws}^{2} & 0\\ \sigma_{x}^{2}-b_{s}^{2}\sigma_{ws}^{2} & 0 & \sigma_{x}^{2}-b_{s}^{2}\sigma_{ws}^{2} \end{array}\right)\right).$$

This distribution yields the calibration model $E\left(X_{sji}|W_{sji}\right)=a_{s}+b_{s}W_{sji}$ and $Cov(W_{sji},$$\epsilon_{sji})=0$. The data were generated for each stratum of each study first, and then a case and a control were randomly chosen in each stratum. In the simulation, we set $\mu_{x}=0$ and $\sigma_{x}^{2}=1$. We assumed four studies in the pooled analysis with 500 case–control pairs (i.e., 1000 total subjects) in each study, and the calibration parameters were set to be $a=\left[-3,1,-1,3\right]$ and $b=\left[0.5,0.75,1.25,1.5\right]$. We set $Var\left(W_{sji}\right)=\sigma_{ws}^{2}=\left[3.8,1.7,0.6,0.4\right]$ to ensure a wide range of variation in local laboratory measurements. The stratum-specific intercept $\beta_{0sj}$ was assumed to follow a normal distribution with a mean of 0 and a variance of 0.01, which was chosen to save computer time in the data generation process.

The spline functions were chosen to be restricted cubic splines and for presentational simplicity, we chose three knots, fixed at the (25th, 50th, 75th) quantiles of N(0,1). We assumed the following risk model without additional covariates:

$$\mathrm{logit}\left(P(Y_{sji}=1|X_{sji})\right)=\beta_{0sj}+\beta_{X_{1}}f_{1}\left(X_{sji}\right)+\beta_{X_{2}}f_{2}\left(X_{sji}\right),$$

where $f_{1}\left(X_{sji}\right)=X_{sji}$, $f_{2}\left(X_{sji}\right)={\left(X_{sji}-t_{1}\right)}_{+}^{3}-{\left(X_{sji}-t_{2}\right)}_{+}^{3}\frac{t_{3}-t_{1}}{t_{3}-t_{2}}+{\left(X_{sji}-t_{3}\right)}_{+}^{3}\frac{t_{2}-t_{1}}{t_{3}-t_{2}}$, and $t_{1},t_{2},t_{3}$ are the three knots mentioned above [18]. Note that $\beta_{X_{2}}=0$ implies a linear relationship between the biomarker level and the log relative risk of disease.

The simulations were performed 1000 times for different combinations of $\left(\beta_{X_{1}},\beta_{X_{2}}\right)$, and calibration proportions, defined as the proportion of controls re-assayed in the reference laboratory. We set $|\beta_{X_{1}}|$ at relatively large values to evaluate the performance of our method, even when the effect of the biomarker is relatively strong, and $\beta_{X_{2}}$ was determined so that the nonlinear relationships across the ranges of $\beta_{X_{1}}$ considered in the tables is moderate. The calibration proportions were 5%, 10%, or 30% in each contributing study, which represents a reasonable range for the size of the calibration subsets in practice.

We compared the performance of both the internalized (IN) and full (FC) calibration methods in terms of percent bias ($\frac{\hat{\beta }-\beta }{\beta }\times 100\%$) over the simulation replicates and coverage rate, which is defined as the proportion of the estimated 95% confidence intervals containing the true value. We also included the naive (N) method for comparison, where no calibration was performed, and the conditional logistic regression was fitted using the local laboratory measurements directly.

The simulation results in Table 1 and Table 2 are for a biomarker that had an inverse effect on the disease risk and the Supplementary Tables S1 and S2 in Supplementary Materials are for a biomarker that had a positive association with the disease risk. The naive method performed poorly in all scenarios regardless of the calibration proportions. The percent biases of the naive estimates were typically larger than 30%, and the coverage rates were typically below 70%. The internalized and full calibration estimates had consistently better performance than the naive method. Full calibration estimates were robust over many combinations of coefficients and calibration proportions, where the percent biases were typically below 10% when the calibration proportion was 5% and below 5% when calibration proportions were 15% and 30%. The coverage rates ranged from 93% to 97%, which were close to the 95% nominal level. The internalized calibration estimates were less robust than the full calibration estimates, and they tended to be more biased when the calibration proportion was large. Section S4 of the Supplementary Materials contains a mathematical justification for the relative performance of the internalized and full calibration methods.

**Figure 1:**
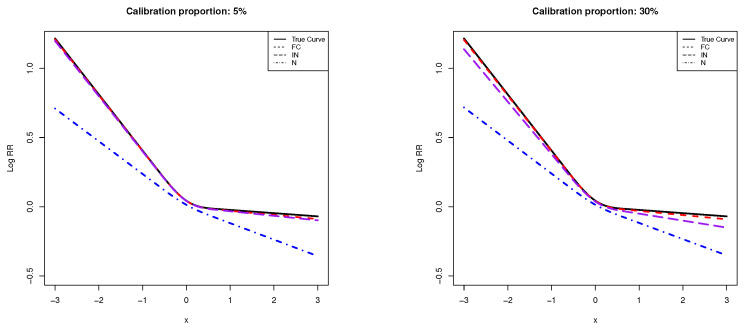
The average of the dose–response curves over 1000 simulations. X-axis is the biomarker measurement, and y-axis is the log RR of the disease. The solid line is the true curve, and the dotted and dashed lines were estimated using internalized (IN) and full calibration methods (FC), respectively, while the dashed-dotted line is estimated using the naive method (N). The calibration proportion is 5% (left) and 30% (right), and the coefficients of the spline functions are set to be −log(1.5)≈−0.41 and 0.14, respectively.

We also plotted the curves reflecting the biomarker-disease association. The x-axis represents the biomarker values, and the y-axis is the log RR. Figure 1 is for the scenario where $\beta_{X_{1}}=-\log\left(1.5\right)\approx -0.41$ and $\beta_{X_{2}}=0.14$, and the calibration proportions were set to be 5% and 30%, respectively. We can see that the curve estimated using the naive method deviated from the true curve substantially, while the curves estimated using the internalized and full calibration methods were closer to the true curve. As the calibration proportion increased, the curve estimated using the full calibration method was closer to the true curve than the internalized calibration method. Supplementary Figure S1 in the Supplementary Materials is for the scenario when $\beta_{X_{1}}=\log\left(1.75\right)\approx 0.56$ and $\beta_{X_{2}}=-0.16$, and the calibration proportions were also set to be 5% and 30%, respectively. We can see similar behaviors of the three estimated curves, where the full calibration method led to the estimated curve that was closest to the true curve and was robust over all calibration proportions.

In addition, we changed $\sigma_{ws}^{2}$ in the simulation setup to vary the ratio of $\frac{\sigma_{ws}^{2}}{\sigma_{x}^{2}}$. This ratio for each study was chosen among 0.75, 0.85, 0.90, and 0.95. The simulation results in Table 3 shows that the performance of the calibration models improved when this variance ratio increased; that is, when the error term in the calibration model X|W became smaller. The full calibration method was more robust than the internalized calibration method with smaller percent biases and coverage rates closer to the 95% nominal level for all the variance ratios considered.

Lastly, we set the coefficient of the nonlinear term to 0 (i.e., $\beta_{X_{2}}=0$) and evaluated the performance of our methods for testing the null hypothesis of no nonlinear effect. Simulation results are reported in Supplementary Tables S4 and S5. Both the full and internalized calibration methods have coverage rates of 95% confidence intervals close to 95% for $\beta_{X_{2}}=0$, suggesting that when there is no nonlinear effect, the Wald test based on the point and variance estimates in the full and internalized calibration methods will have a type-I error rate close to 0.05. However, the naive method has coverage rates ranging from 8% to 70%, indicating that directly pooling biomarker measurements together without calibration may lead to false positive evidence for a nonlinear effect.

## 4. Applied Example

We applied our methods to evaluate the association of 25(OH)D with colorectal cancer incidence. This example was based on two large cohort studies in the United States: Nurses’ Health Study (NHS) [22] and Health Professionals Follow-up Study (HPFS) [23]. In the NHS, 121,701 female nurses aged 30 to 55 were enrolled in 1976, while in the HPFS, 51,529 male health professionals aged 40 to 75 were enrolled in 1986. To account for assay differences or laboratory drift over time, within each study, all measurements were calibrated to a common assay prior to the analyses. In all, our pooling analysis consisted of 1876 subjects with a nested case–control study design. The matching factors mainly included age at blood collection and date of blood collection. For the calibration subsets, controls in each study were divided into 10 deciles based on the 25(OH)D levels, and three subjects were randomly sampled in each decile, except for one decile, where only two subjects were selected [4]. A total of twenty-nine controls in each nested case–control study were selected to have their blood samples re-assayed at Heartland Assays, LLC (Ames, IA, USA), the reference laboratory, from 2011 to 2013. We refer readers to the paper by McCullough et al. [4] for detailed patient selection criteria.

Table 4 presents sample sizes and parameter estimates along with standard errors of the study-specific calibration models. The potential confounders that were adjusted for in the conditional logistic regression model included smoking (yes/no), BMI (greater or less than 25), physical activity (continuous), and family history of myocardial infarction (yes/no). We used the restricted cubic spline with three knots at the 25%, 50%, and 75% quantiles of the reference 25(OH)D measurements to estimate how the log RR changes with the Vitamin D levels. Table 5 presents the coefficient estimates along with corresponding 95% confidence intervals.

As shown in Table 5, we obtained similar point and confidence interval estimates of coefficients from the internalized and full calibration methods. We observed a significant linear relationship between 25(OH)D measurements and log RR of colorectal cancer (*p*-value = 0.0211 and 0.0217, for the internalized and full calibration methods, respectively), while the nonlinear term was not significant (*p*-value = 0.2162 and 0.2219, for the internalized and full calibration methods, respectively). Therefore, we concluded that circulating Vitamin D level had a significant linear association with the log relative risk of colorectal cancer.

After dropping the nonlinear term from the conditional logistic regression model and refitting the model using the linear method proposed by Sloan et al. [12], in Table 5, the point estimate of the biomarker effect on the log RR of colorectal cancer based on full calibration method was −0.0059 (RR=0.9941) for a 1 nmol/L increase in 25(OH)D, with a *p*-value of 0.0177, and the 95% confidence interval was (−0.0108,−0.0010), suggesting a significant negative linear relationship between levels of circulating 25(OH)D measurements and the log relative risk of colorectal cancer.

**Figure 2:**
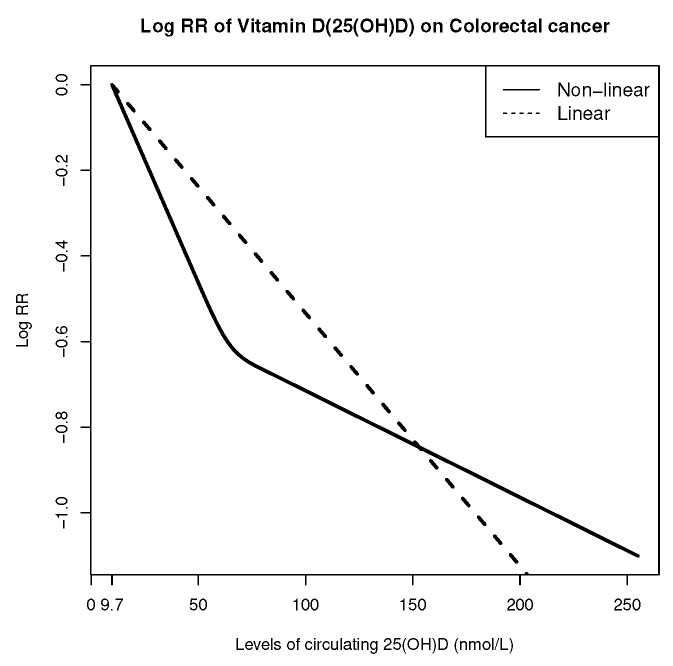
Log colorectal cancer RR for levels of circulating 25(OH)D compared to the reference level, 9.734 nmol/L, based on the full calibration method.

In Figure 2, we plotted the log RR of colorectal cancer on circulating 25(OH)D measurements under both models with and without the nonlinear spline term. We set the reference level to be individuals with the minimum 25(OH)D measurement 9.734 nmol/L in the aggregated study.

## 5. Discussion

In this paper, we propose statistical methods for analyzing pooled matched/nested case–control studies. To apply our method, an assumption is that, for each study, the relationship between the measurements from the local laboratory and those from the reference laboratory estimated in the calibration subset represents that in the entire study. This assumption is likely to be violated for laboratories that do not follow good laboratory practices, even if the calibration subset is selected randomly. Our methods can estimate a possibly nonlinear dose–response curve between biomarker measurements and the diseases and can evaluate whether the relationship is linear or not. We focus on the common situation in which only controls are selected for re-assaying in the reference laboratory. We derived an analytic expression for the variance–covariance matrix of the estimated coefficients in the conditional logistic regression model that takes account of the uncertainty from fitting the study-specific calibration models.

Several remarks and recommendations can be drawn from our work. The full calibration method led to estimates with smaller percent biases in all simulation scenarios, and coverage rates were closer to the 95% nominal level. As the calibration proportion increased, the internalized calibration method became more biased than the full calibration method. Since the calibration model was fitted among controls only, estimates of the model parameters were slightly biased. The bias in the intercept was canceled out in the approximate likelihood function in the full calibration method but not in the internalized calibration method. Therefore, we recommend using the full calibration method for analyses that require the calibration of local laboratory measurements to a reference laboratory. When designing a new study, as discussed in Sloan et al. [15], the calibration set sample size should be sufficiently large so that the estimates in the calibration models are stable. Based on our simulation study, the full calibration method can perform reasonably well when the calibration proportion is at least 5% in studies with 500 case–control pairs and the laboratory errors are moderate.

Our method can be used to estimate dose–response curves between biomarkers and disease risks. Based on the proposed analytic variance estimators that account for the calibration process, the method can also be used to perform statistical tests to evaluate the existence of nonlinear trends. When, in fact, $\beta_{X_{2}}=0$, the estimated coefficient of the linear term from our method including both linear and nonlinear terms should be similar to that from the linear model method by Sloan et al. [12], but the variance in the estimated coefficient of the linear term from our model may be slightly larger than that from the model including the linear term alone due to estimating additional coefficients for the nonlinear terms in our method. If there is not enough evidence to reject the null hypothesis of no nonlinear effects, we recommend re-fitting the model using the linear method proposed by Sloan et al. [12].

The R code for pooling matched/nested case–control study using restricted cubic spline functions is available at https://www.hsph.harvard.edu/molin-wang/section-3-pooling-biomarker-data/ (accessed on 1 April 2022).

## Figures and Tables

**Table 1:** Comparison of operating characteristics for $\beta_{X}$ under the model $P\left(Y_{sji}=1|X_{sji}\right)=\beta_{0sj}+\beta_{X_{1}}f_{1}\left(X_{sji}\right)+\beta_{X_{2}}f_{2}\left(X_{sji}\right)$ for Internalized calibration (IN), Full calibration (FC), and Naive methods (N). Relative bias is computed using $\frac{\hat{\beta }-\beta }{\beta }$, and the reported value in the table is the average over the 1000 simulation replicates. Coverage rate is the proportion of simulations that yield a 95% confidence interval covering the true parameter. Standard deviation is the square root of the empirical variance of parameter estimates over all replicates; we report $10^{3}$ times the standard deviation. The calibration proportions (denoted as Calib. size in the table) were set to be 5%, 15%, and 30%. $\beta_{X_{2}}$ is fixed at 0.08.

Calib. Size	$\beta_{X_{1}}$	Relative Bias of $\beta_{X_{1}}$ (SD)			Coverage Rate of $\beta_{X_{1}}$			Relative Bias of $\beta_{X_{2}}$ (SD)			Coverage Rate of $\beta_{X_{2}}$		
		IN	FC	N	IN	FC	N	IN	FC	N	IN	FC	N
5%	−log(1.25)	−1.6% (2.621)	−0.1% (2.959)	−44.4% (0.110)	0.970	0.972	0.458	−3.6% (0.192)	−0.6% (0.196)	−72.2% (0.018)	0.968	0.971	0.222
	−log(1.5)	−1.2% (2.601)	−0.3% (2.972)	−23.7% (0.160)	0.964	0.966	0.518	−5.2% (0.220)	−2.1% (0.228)	−36% (0.025)	0.962	0.962	0.736
	−log(1.75)	−1.1% (2.846)	−0.4% (3.288)	−16.6% (0.207)	0.964	0.966	0.569	−8.5% (0.258)	−5.3% (0.257)	−7.6% (0.033)	0.957	0.959	0.941
	−log(2)	−1.4% (2.828)	−0.8% (3.212)	−12.8% (0.261)	0.968	0.970	0.642	−11.9% (0.337)	−8.7% (0.339)	18.2% (0.040)	0.955	0.958	0.888
	−log(2.25)	−1% (3.707)	−0.5% (4.481)	−10% (0.324)	0.975	0.980	0.707	−10.8% (0.357)	−7.5% (0.371)	41.1% (0.051)	0.954	0.955	0.766
	−log(2.5)	−1.1% (3.785)	−0.6% (4.486)	−8.2% (0.405)	0.961	0.963	0.754	−12% (0.385)	−8.5% (0.393)	62% (0.066)	0.944	0.944	0.609
	−log(2.75)	−1% (4.444)	−0.6% (5.136)	−7.2% (0.452)	0.965	0.963	0.788	−12% (0.517)	−8.7% (0.535)	78% (0.075)	0.954	0.954	0.497
15%	−log(1.25)	−7.1% (0.694)	−2.3% (0.874)	−44.1% (0.120)	0.966	0.969	0.476	−13.6% (0.095)	−4.3% (0.096)	−70.8% (0.019)	0.944	0.953	0.267
	−log(1.5)	−3.9% (0.854)	−1.1% (1.134)	−24.2% (0.154)	0.957	0.956	0.511	−13.4% (0.115)	−3.8% (0.115)	−36.4% (0.024)	0.947	0.952	0.739
	−log(1.75)	−2.4% (0.836)	−0.3% (1.123)	−17.1% (0.197)	0.955	0.962	0.564	−12.8% (0.145)	−2.9% (0.147)	−8.8% (0.031)	0.941	0.950	0.937
	−log(2)	−1.8% (0.911)	0% (1.243)	−12.7% (0.265)	0.961	0.966	0.638	−11.9% (0.174)	−1.9% (0.175)	19.4% (0.043)	0.951	0.949	0.897
	−log(2.25)	−1.4% (1.218)	0.3% (1.797)	−9.7% (0.326)	0.961	0.972	0.722	−14.9% (0.215)	−4.6% (0.217)	42.1% (0.051)	0.944	0.947	0.765
	−log(2.5)	−2.1% (1.108)	−0.6% (1.595)	−8.3% (0.395)	0.941	0.948	0.761	−18.4% (0.247)	−7.6% (0.249)	62.0% (0.064)	0.938	0.950	0.620
	−log(2.75)	−1.4% (1.273)	0.1% (1.926)	−6.7% (0.461)	0.948	0.960	0.805	−16.9% (0.295)	−6.1% (0.299)	79.7% (0.076)	0.941	0.950	0.481
30%	−log(1.25)	−9.9% (0.519)	−0.3% (0.455)	−43.3% (0.113)	0.943	0.955	0.477	−21.1% (0.083)	−2.3% (0.084)	−70.8% (0.019)	0.937	0.954	0.265
	−log(1.5)	−5.1% (0.514)	0.6% (0.561)	−23.2% (0.164)	0.944	0.951	0.533	−19.7% (0.096)	−0.5% (0.096)	−35.9% (0.026)	0.948	0.957	0.751
	−log(1.75)	−4.5% (0.564)	−0.2% (0.659)	−16.7% (0.211)	0.940	0.960	0.576	−23.6% (0.129)	−4.1% (0.129)	−7.7% (0.034)	0.941	0.962	0.926
	−log(2)	−4.0% (0.581)	−0.3% (0.755)	−12.8% (0.258)	0.937	0.961	0.631	−25.5% (0.156)	−5.4% (0.155)	18.7% (0.042)	0.936	0.956	0.900
	−log(2.25)	−3.6% (0.661)	−0.3% (0.885)	−10.1% (0.328)	0.926	0.931	0.698	−28.1% (0.193)	−7.4% (0.193)	41.1% (0.052)	0.922	0.933	0.769
	−log(2.5)	−3.1% (0.719)	0.1% (0.988)	−8.3% (0.409)	0.939	0.954	0.749	−24.2% (0.233)	−3% (0.234)	62.1% (0.065)	0.938	0.948	0.613
	−log(2.75)	−3.5% (0.835)	−0.6% (1.109)	−7.2% (0.459)	0.926	0.954	0.794	30.1% (0.274)	−8.9% (0.272)	78.6% (0.076)	0.910	0.943	0.506

**Table 2:** Comparison of operating characteristics for $\beta_{X}$ under the model $P\left(Y_{sji}=1|X_{sji}\right)=\beta_{0sj}+\beta_{X_{1}}f_{1}\left(X_{sji}\right)+\beta_{X_{2}}f_{2}\left(X_{sji}\right)$ for Internalized calibration (IN), Full calibration (FC), and Naive methods (N). Relative bias is computed using $\frac{\hat{\beta }-\beta }{\beta }$, and the reported value in the table is the average over the 1000 simulation replicates. Coverage rate is the proportion of simulations that yield a 95% confidence interval covering the true parameter. Standard deviation is the square root of the empirical variance of parameter estimates over all replicates; we report $10^{3}$ times the standard deviation. The calibration proportions (denoted as Calib. size in the table) were set to be 5%, 15%, and 30%. $\beta_{X_{1}}$ is fixed at −log(1.5).

Calib. Size	$\beta_{X_{2}}$	Relative Bias of $\beta_{X_{1}}$ (SD)			Coverage Rate of $\beta_{X_{1}}$			Relative Bias of $\beta_{X_{2}}$ (SD)			Coverage Rate of $\beta_{X_{2}}$		
		IN	FC	N	IN	FC	N	IN	FC	N	IN	FC	N
5%	0.02	−1.3% (2.838)	−0.4% (3.140)	−6.5% (0.178)	0.968	0.970	0.898	−21.6% (0.215)	−9.3% (0.218)	188.8% (0.029)	0.962	0.958	0.624
	0.06	−2.2% (3.199)	−1.2% (3.528)	−18.1% (0.159)	0.960	0.967	0.686	−12.9% (0.234)	−8.8% (0.247)	−12.4% (0.025)	0.949	0.954	0.938
	0.10	−2.5% (2.515)	−1.6% (2.956)	−29.5% (0.144)	0.968	0.969	0.339	−8.1% (0.197)	−5.6% (0.206)	50.8% (0.023)	0.959	0.957	0.399
	0.14	−1.5% (2.815)	−0.6% (3.179)	−41.7% (0.131)	0.967	0.970	0.078	−4.1% (0.194)	−2.3% (0.198)	−69.3% (0.021)	0.953	0.951	0.010
	0.18	−2.1% (2.636)	−1.2% (3.149)	−52.8% (0.127)	0.968	0.969	0.012	−5.0% (0.219)	−3.6% (0.225)	−78.0% (0.020)	0.950	0.954	0.000
15%	0.02	−2.7% (0.889)	0.1% (1.072)	−5.8% (0.176)	0.951	0.953	0.922	−49.7% (0.126)	−11.7% (0.126)	191.9% (0.027)	0.928	0.938	0.630
	0.06	−3.3% (0.771)	−0.5% (0.994)	−17.7% (0.161)	0.954	0.970	0.699	−19.0% (0.122)	−6.2% (0.123)	−11.6% (0.026)	0.938	0.948	0.933
	0.10	−3.3% (0.867)	−0.5% (0.982)	−29.8% (0.143)	0.942	0.944	0.335	−10.3% (0.114)	−2.6% (0.113)	−52.1% (0.023)	0.933	0.948	0.376
	0.14	−3.7% (0.790)	−0.9% (1.059)	−41.8% (0.136)	0.952	0.961	0.083	−7.9% (0.113)	−2.3% (0.114)	−69.2% (0.022)	0.954	0.962	0.008
	0.18	−4.3% (0.851)	−1.4% (1.163)	−53.6% (0.118)	0.959	0.964	0.003	−7.2% (0.108)	−2.7% (0.109)	−78.5% (0.019)	0.956	0.958	0.000
30%	0.02	−6.0% (0.546)	−0.5% (0.569)	−6.5% (0.175)	0.945	0.963	0.916	−85.6% (0.110)	−10.4% (0.111)	189.7% (0.028)	0.932	0.957	0.640
	0.06	−5.6% (0.556)	0.0% (0.583)	−18.0% (0.162)	0.939	0.955	0.682	−28.5% (0.101)	−2.9% (0.101)	−12.3% (0.026)	0.926	0.952	0.928
	0.10	−6.1% (0.536)	−0.4% (0.619)	−29.4% (0.151)	0.927	0.943	0.342	−19.2% (0.097)	−3.6% (0.097)	−51.7% (0.024)	0.929	0.942	0.369
	0.14	−6.4% (0.458)	−0.8% (0.528)	−41.1% (0.135)	0.951	0.954	0.080	−13.9% (0.092)	−2.7% (0.091)	−68.2% (0.022)	0.927	0.953	0.011
	0.l8	−6.9% (0.493)	−1.2% (0.618)	−53.6% (0.125)	0.935	0.957	0.006	−11.8% (0.090)	−2.8% (0.091)	−78.6% (0.020)	0.920	0.950	0.000

**Table 3:** Comparison of operating characteristics for $\beta_{X}$ under the model $P\left(Y_{sji}=1|X_{sji}\right)=\beta_{0sj}+\beta_{X_{1}}f_{1}\left(X_{sji}\right)+\beta_{X_{2}}f_{2}\left(X_{sji}\right)$ for Internalized calibration (IN), Full calibration (FC), and Naive methods (N) with different $\frac{\sigma_{ws}^{2}}{\sigma_{X}^{2}}$. Relative bias is computed using $\frac{\hat{\beta }-\beta }{\beta }$, and the reported value is the average over the 1000 simulation replicates. Coverage rate is the proportion of simulations that yield a 95% confidence interval covering the true parameter. Standard deviation is the square root of the empirical variance of parameter estimates over all replicates; we report $10^{3}$ times the standard deviation. The calibration proportions (denoted as Calib. size in the table) were set to be 5%, 15%, and 30%. $\beta_{X_{1}}=-0.25,\beta_{X_{2}}=0.08$.

Calib. Size	$\frac{\sigma_{\mathrm{ws}}^{2}}{\sigma_{x}^{2}}$	Relative Bias of $\beta_{X_{1}}$ (SD)			Coverage Rate of $\beta_{X_{1}}$			Relative Bias of $\beta_{X_{2}}$ (SD)			Coverage Rate of $\beta_{X_{2}}$		
		IN	FC	N	IN	FC	N	IN	FC	N	IN	FC	N
5%	0.75	−14.1% (22.094)	−5.1% (27.250)	−40.6% (0.145)	0.970	0.983	0.509	−35.0% (1.966)	−15.2% (2.444)	−69.5% (0.023)	0.942	0.968	0.375
	0.85	−9.0% (8.468)	−4.4% (9.954)	−40.9% (0.128)	0.966	0.976	0.479	−20.5% (0.700)	−10.4% (0.800)	−68.2% (0.020)	0.944	0.958	0.364
	0.90	−5.3% (4.876)	−2.5% (5.373)	−40.5% (0.125)	0.967	0.976	0.460	−10.6% (0.316)	−4.4% (0.336)	−67.0% (0.020)	0.943	0.954	0.338
	0.95	−1.5% (2.398)	−0.2% (2.626)	−38.0% (0.123)	0.950	0.953	0.504	−4.7% (0.184)	−1.7% (0.187)	−64.7% (0.019)	0.947	0.953	0.352
15%	0.75	−31.1% (5.298)	−3.9% (6.133)	−40.7% (0.151)	0.908	0.976	0.528	−71.3% (0.392)	−11.7% (0.420)	−68.9% (0.024)	0.808	0.957	0.383
	0.85	−15.6% (2.250)	−1.8% (2.826)	−39.8% (0.135)	0.954	0.975	0.500	−35.4% (0.200)	−5.0% (0.213)	−67.0% (0.021)	0.927	0.966	0.344
	0.90	−10.4% (1.456)	−1.7% (1.794)	−38.7% (0.124)	0.951	0.960	0.504	−24.6% (0.145)	−5.4% (0.152)	−65.5% (0.020)	0.933	0.960	0.351
	0.95	−4.5% (0.732)	−0.5% (0.838)	−38.6% (0.130)	0.961	0.961	0.476	−11.4% (0.097)	−2.5% (0.098)	−65.6% (0.020)	0.949	0.960	0.318
30%	0.75	−58.0% (3.127)	−4.4% (3.189)	−40.3% (0.144)	0.639	0.977	0.547	−130.8% (0.209)	−13.4% (0.224)	−68.3% (0.023)	0.390	0.951	0.379
	0.85	−31.6% (1.467)	−4.0% (1.350)	−40.1% (0.132)	0.837	0.967	0.494	−70.5% (0.128)	−9.8% (0.131)	−67.3% (0.021)	0.780	0.951	0.361
	0.90	−18.9% (0.913)	−1.7% (1.008)	−39.1% (0.127)	0.91	0.961	0.478	−42.8% (0.105)	−4.9% (0.108)	−66.4% (0.020)	0.891	0.957	0.336
	0.95	−9.5% (0.475)	−1.4% (0.456)	−37.7% (0.122)	0.924	0.935	0.492	−22.9% (0.083)	−5.1% (0.084)	−64.0% (0.019)	0.920	0.935	0.336

**Table 4:** Number of cases and controls, size of the calibration study ($n_{cal}$), and the estimated intercept and slope of the calibration model for each study in the pooled analysis.

Study	Cases/Controls	$n_{\mathrm{cal}}$	$\hat{a}$ (SE)	$\hat{b}$ (SE)
NHS	348/694	29	−3.56 (2.72)	1.13 (0.97)
HPFS	267/519	29	3.38 (2.95)	0.05 (0.04)

**Table 5:** Point estimates and 95% confidence intervals for the nonlinear (and linear) association of circulating 25(OH)D (nmol/L) with colorectal cancer after adjusting for BMI (overweight or not), physical activity (continuous), smoking (never/ever), and family history of colorectal cancer (yes/no).

Method	$\beta_{X_{1}}$	$\beta_{X_{2}}$
Internalized calibration	−0.0116 (−0.0214, −0.0017)	7.9307$\times 10^{-6}$ (−4.6375$\times 10^{-6}$, 2.0499$\times 10^{-5}$)
Full calibration	−0.0115 (−0.0213, −0.0017)	7.9885$\times 10^{-6}$ (−4.8290$\times 10^{-6}$, 2.0806$\times 10^{-5}$)
Linear model (FC)	−0.0059 (−0.0108, −0.0010)	-

## Data Availability

The datasets used in the real data analysis are not publicly available. Further information including the procedures to obtain and access data from the Nurses’ Health Studies and the Health Professionals Follow-up Study is described at https://www.nurseshealthstudy.org/researchers/ and https://sites.sph.harvard.edu/hpfs/for-collaborators/ (accessed on 1 April 2022).

## Supplementary Materials

The following supporting information can be downloaded at: https://www.mdpi.com/article/10.3390/cancers14010000/s1. Supplementary Table S1: $\beta_{X_{2}}=-0.16$; Supplementary Table S2: $\beta_{X_{1}}=\log\left(1.5\right)$; Supplementary Table S3: $\beta_{X_{1}}=0.48,\beta_{X_{2}}=-0.16$; Supplementary Tables S4 and S5: We set the nonlinear effect to be 0 (i.e., $\beta_{X_{2}}=0$). Supplementary Figure S1: Curves reflecting the association of biomarker measurements on disease risk when $\beta_{X_{1}}=\log\left(1.75\right),\beta_{X_{2}}=-0.16$.
