# Supplementary material for: Spline Analysis of Biomarker Data Pooled from Multiple Matched/Nested Case–Control Studies
Source: Cancers (Basel). 2022 Jun 3;14(11):2783. doi: 10.3390/cancers14112783 (PMC9179317; doi:10.3390/cancers14112783)
Supplement: Supplementary file 1 [file cancers-14-02783-s001.zip › cancers-1691890-supplementary.pdf]

Spline analysis of biomarker data pooled from multiple matched/nested case-control studies—Supplementary Material

S1 Approximate Likelihood Derivation

Let vectors $\boldsymbol{X}_{sj}$, $\boldsymbol{W}_{sj}$ and matrix $\boldsymbol{Z}_{sj}$ contain measurement of individuals from the jth stratum of sth study. The conditional likelihood contribution for the jth stratum of the sth study is:

$$
\begin{aligned}
L^*_{sj} &= P(Y_{sj1}=0,\ldots,Y_{sj,m_{sj}}=0,Y_{sj,m_{sj}+1}=1,\ldots,Y_{sj,m_{sj}+n_{sj}}=1|\boldsymbol{X}_{sj},\boldsymbol{Z}_{sj},\sum_{i=1}^{m_{sj}+n_{sj}} Y_{sji}=n_{sj})\\
&= \frac{\prod_{l=1}^{n_{sj}} \exp\left(\beta_{0sj}+\boldsymbol{\beta}_X^T \boldsymbol{f}(X_{sj,m_{sj}+l})+\boldsymbol{\beta}_{\mathbf{Z}}^T \mathbf{Z}_{sj,m_{sj}+l}\right)}{\sum_{(i_1,\ldots,i_{n_{sj}})\in A_{sj}} \prod_{l=1}^{n_{sj}} \exp(\beta_{0sj}+\boldsymbol{\beta}_X^T \boldsymbol{f}(X_{sj,i_l})+\boldsymbol{\beta}_{\mathbf{Z}}^T \mathbf{Z}_{sj,i_l})}\\
&= \left(1+\sum_{(i_1,\ldots,i_{n_{sj}})\in A'_{sj}} \exp\left(\boldsymbol{\beta}_X^T \sum_{l=1}^{n_{sj}}\left[\boldsymbol{f}(X_{sj,i_l})-\boldsymbol{f}(X_{sj,m_{sj}+l})\right]+\boldsymbol{\beta}_{\mathbf{Z}}^T \sum_{l=1}^{n_{sj}}\left[\mathbf{Z}_{sj,i_l}-\mathbf{Z}_{sj,m_{sj}+l}\right]\right)\right)^{-1}
\end{aligned} \tag{1}
$$

where A_{sj} is the set of all subsets of indices of size n_{sj} of the set $\{1,2,\ldots,m_{sj}, m_{sj}+1,\ldots,m_{sj}+n_{sj}\}$ and $\{i_1,i_2,\ldots,i_{n_{sj}}\}$ corresponds to one specific such subset of size n_{sj}. A'_{sj} is the subset of A_{sj} that excludes subset where $i_1=m_{sj}+1, i_2=m_{sj}+2,\ldots,i_{n_{sj}}=m_{sj}+n_{sj}$.

Under the surrogacy assumption

$$
P(\mathbf{Y}_{sj}|\mathbf{X}_{sj},\mathbf{W}_{sj},\mathbf{Z}_{sj},\sum_{i=1}^{n_{sj}+m_{sj}} Y_{sji}=n_{sj}) = P(\mathbf{Y}_{sj}|\mathbf{X}_{sj},\mathbf{Z}_{sj},\sum_{i=1}^{n_{sj}+m_{sj}} Y_{sji}=n_{sj})
$$

the likelihood contributed from a stratum using local laboratory biomarker measurements is:

$$
\begin{aligned}
L_{sj} =& P\left(Y_{sj1}=0,\ldots,Y_{sj,m_{sj}}=0,Y_{sj,m_{sj}+1}=1,\ldots,Y_{sj,m_{sj}+n_{sj}}=1|\boldsymbol{W}_{sj},\boldsymbol{Z}_{sj},\sum_{i=1}^{m_{sj}+n_{sj}}Y_{sji}=n_{sj}\right)\\
=& \int P\left(Y_{sj1}=0,\ldots,Y_{sj,m_{sj}}=0,Y_{sj,m_{sj}+1}=1,\ldots,Y_{sj,m_{sj}+n_{sj}}=1|\boldsymbol{X}_{sj},\boldsymbol{W}_{sj},\boldsymbol{Z}_{sj},\sum_{i=1}^{m_{sj}+n_{sj}}Y_{sji}=n_{sj}\right)\\
&\times P\left(\boldsymbol{X}_{sj}|\boldsymbol{W}_{sj},\boldsymbol{Z}_{sj},\sum_{i=1}^{m_{sj}+n_{sj}}Y_{sji}=n_{sj}\right)d\boldsymbol{X}_{sj}\\
=& \int P\left(Y_{sj1}=0,\ldots,Y_{sj,m_{sj}}=0,Y_{sj,m_{sj}+1}=1,\ldots,Y_{sj,m_{sj}+n_{sj}}=1|\boldsymbol{X}_{sj},\boldsymbol{Z}_{sj},\sum_{i=1}^{m_{sj}+n_{sj}}Y_{sji}=n_{sj}\right)\\
&\times P\left(\boldsymbol{X}_{sj}|\boldsymbol{W}_{sj},\boldsymbol{Z}_{sj},\sum_{i=1}^{m_{sj}+n_{sj}}Y_{sji}=n_{sj}\right)d\boldsymbol{X}_{sj}\\
=& \int\left(1+\sum_{(i_1,\ldots,i_{n_{sj}})\in A'_{sj}}\exp\left(\boldsymbol{\beta}_X^T\sum_{l=1}^{n_{sj}}\left[\boldsymbol{f}(X_{sj,i_l})-\boldsymbol{f}(X_{sj,m_{sj}+l})\right]+\boldsymbol{\beta}_{\mathbf{Z}}^T\sum_{l=1}^{n_{sj}}\left[\mathbf{Z}_{sj,i_l}-\mathbf{Z}_{sj,m_{sj}+l}\right]\right)\right)^{-1}\\
&\times P\left(\boldsymbol{X}_{sj}|\boldsymbol{W}_{sj},\boldsymbol{Z}_{sj},\sum_{i=1}^{m_{sj}+n_{sj}}Y_{sji}=n_{sj}\right)d\boldsymbol{X}_{sj}\\
=& E_{\boldsymbol{X}_{sj}|\boldsymbol{W}_{sj},\boldsymbol{Z}_{sj},\sum_{i=1}^{n_{sj}+m_{sj}}Y_{sji}=n_{sj}}\left(L^*_{sj}\right)
\end{aligned}
$$

where L^*_{sj} is defined in Equation (1), and we use surrogacy assumption in the third line.

We approximate the likelihood L^*_{sj} contributed from the jth stratum of sth study using a second order Taylor expansion with respect to $\mathbf{X}_{sj}$ at $\widetilde{\boldsymbol{X}}_{sj} = E\left(\boldsymbol{X}_{sj}|\boldsymbol{W}_{sj},\boldsymbol{Z}_{sj},\sum_{i=1}^{m_{sj}+n_{sj}}Y_{sji}=n_{sj}\right)$:

$$
\begin{aligned}
L^*_{sj} \approx\ & L^*_{sj}|_{\boldsymbol{X}_{sj}=\widetilde{\boldsymbol{X}}_{sj}} \\
& - \sum_{i=1}^{m_{sj}} \frac{\sum_{(i_1,\ldots,i_{n_{sj}})\in B_{sj}} \left[\exp\left(G_{i_l}\right)\boldsymbol{\beta}_X^T \boldsymbol{f'}(X_{sji})\right]}{(1+\sum_{(i_1,\ldots,i_{n_{sj}})\in A'_{sj}} G_{i_l})^2}|_{\boldsymbol{X}_{sj}=\widetilde{\boldsymbol{X}}_{sj}} \Delta_{sji} \\
& - \sum_{i=m_{sj}+1}^{m_{sj}+n_{sj}} \frac{\sum_{(i_1,\ldots,i_{n_{sj}})\in C_{sj}} \left[\exp\left(G_{i_l}\right)(-\boldsymbol{\beta}_X^T \boldsymbol{f'}(X_{sji}))\right]}{(1+\sum_{(i_1,\ldots,i_{n_{sj}})\in A'_{sj}} G_{i_l})^2}|_{\boldsymbol{X}_{sj}=\widetilde{\boldsymbol{X}}_{sj}} \Delta_{sji} \\
& - \frac{1}{2}\sum_{i=1}^{m_{sj}} \frac{\begin{array}{c}\left[\sum_{(i_1,\ldots,i_{n_{sj}})\in B_{sj}} \exp\left(G_{i_l}\right)(\boldsymbol{\beta}_X^T \boldsymbol{f'}(X_{sji}))^2 + \sum_{(i_1,\ldots,i_{n_{sj}})\in B_{sj}} \exp(G_{i_l})\boldsymbol{\beta}_X^T \boldsymbol{f''}(X_{sji})\right] \\ \times (1+\sum_{(i_1,\ldots,i_{n_{sj}})\in A'_{sj}} G_{i_l}) - 2(\sum_{(i_1,\ldots,i_{n_{sj}})\in B_{sj}} \exp(G_{i_l})\boldsymbol{\beta}_X^T \boldsymbol{f'}(X_{sji}))^2\end{array}}{\left(1+\sum_{(i_1,\ldots,i_{n_{sj}})\in A'_{sj}} G_{i_l}\right)^3}|_{\boldsymbol{X}_{sj}=\widetilde{\boldsymbol{X}}_{sj}} \Delta^2_{sji} \\
& - \frac{1}{2}\sum_{i=m_{sj}+1}^{m_{sj}+n_{sj}} \frac{\begin{array}{c}\left[\sum_{(i_1,\ldots,i_{n_{sj}})\in C_{sj}} \exp\left(G_{i_l}\right)(\boldsymbol{\beta}_X^T \boldsymbol{f'}(X_{sji}))^2 + \sum_{(i_1,\ldots,i_{n_{sj}})\in C_{sj}} \exp(G_{i_l})(-\boldsymbol{\beta}_X^T \boldsymbol{f''}(X_{sji}))\right] \\ \times (1+\sum_{(i_1,\ldots,i_{n_{sj}})\in A'_{sj}} G_{i_l}) - 2(\sum_{(i_1,\ldots,i_{n_{sj}})\in C_{sj}} \exp(G_{i_l})(-\boldsymbol{\beta}_X^T \boldsymbol{f'}(X_{sji})))^2\end{array}}{\left(1+\sum_{(i_1,\ldots,i_{n_{sj}})\in A'_{sj}} G_{i_l}\right)^3}|_{\boldsymbol{X}_{sj}=\widetilde{\boldsymbol{X}}_{sj}} \Delta^2_{sji} \\
& - \sum_{1\le i<i'\le m_{sj}} \frac{\begin{array}{c}\left[\sum_{(i_1,\ldots,i_{n_{sj}})\in B'_{sj}} \exp(G_{i_l})(\boldsymbol{\beta}_X^T \boldsymbol{f'}(X_{sji}))(\boldsymbol{\beta}_X^T \boldsymbol{f'}(X_{sji'}))\right](1+\sum_{(i_1,\ldots,i_{n_{sj}})\in A'_{sj}} G_{i_l}) \\ -2\left(\sum_{(i_1,\ldots,i_{n_{sj}})\in B'_{sj}} \exp(G_{i_l})\boldsymbol{\beta}_x^T \boldsymbol{f'}(X_{sji})\right)\left(\sum_{(i_1,\ldots,i_{n_{sj}})\in B'_{sj}} \exp(G_{i_l})\boldsymbol{\beta}_x^T \boldsymbol{f'}(X_{sji'})\right)\end{array}}{(1+\sum_{(i_1,\ldots,i_{n_{sj}})\in A'_{sj}} G_{i_l})^3}|_{\boldsymbol{X}_{sj}=\widetilde{\boldsymbol{X}}_{sj}} \Delta_{sji}\Delta_{sji'} \\
& - \sum_{m_{sj}+1\le i<i'\le m_{sj}+n_{sj}} \frac{\begin{array}{c}\left[\sum_{(i_1,\ldots,i_{n_{sj}})\in C'_{sj}} \exp(G_{i_l})(\boldsymbol{\beta}_X^T \boldsymbol{f'}(X_{sji}))(\boldsymbol{\beta}_X^T \boldsymbol{f'}(X_{sji'}))\right](1+\sum_{(i_1,\ldots,i_{n_{sj}})\in A'_{sj}} G_{i_l}) \\ -2\left(\sum_{(i_1,\ldots,i_{n_{sj}})\in C'_{sj}} \exp(G_{i_l})\boldsymbol{\beta}_x^T \boldsymbol{f'}(X_{sji})\right)\left(\sum_{(i_1,\ldots,i_{n_{sj}})\in C'_{sj}} \exp(G_{i_l})\boldsymbol{\beta}_x^T \boldsymbol{f'}(X_{sji'})\right)\end{array}}{(1+\sum_{(i_1,\ldots,i_{n_{sj}})\in A'_{sj}} G_{i_l})^3}|_{\boldsymbol{X}_{sj}=\widetilde{\boldsymbol{X}}_{sj}} \Delta_{sji}\Delta_{sji'} \\
& - \sum_{1\le i\le m_{sj}<i'\le m_{sj}+n_{sj}} \frac{\begin{array}{c}\left[\sum_{(i_1,\ldots,i_{n_{sj}})\in D_{sj}} \exp(G_{i_l})(\boldsymbol{\beta}_X^T \boldsymbol{f'}(X_{sji}))(-\boldsymbol{\beta}_X^T \boldsymbol{f'}(X_{sji'}))\right](1+\sum_{(i_1,\ldots,i_{n_{sj}})\in A'_{sj}} G_{i_l}) \\ -2\left(\sum_{(i_1,\ldots,i_{n_{sj}})\in D_{sj}} \exp(G_{i_l})\boldsymbol{\beta}_x^T \boldsymbol{f'}(X_{sji})\right)\left(\sum_{(i_1,\ldots,i_{n_{sj}})\in D_{sj}} \exp(G_{i_l})(-\boldsymbol{\beta}_x^T \boldsymbol{f'}(X_{sji'}))\right)\end{array}}{(1+\sum_{(i_1,\ldots,i_{n_{sj}})\in A'_{sj}} G_{i_l})^3}|_{\boldsymbol{X}_{sj}=\mu(\boldsymbol{X}_{sj})} \Delta_{sji}\Delta_{sji'} \\
& + \text{remainder}
\end{aligned}
$$

where

$G_{i_l} = \boldsymbol{\beta}_X^T \sum_{l=1}^{n_{sj}} \left[\boldsymbol{f}(X_{sj,i_l}) - \boldsymbol{f}(X_{sj,m_{sj}+l})\right] + \boldsymbol{\beta}_{\boldsymbol{Z}}^T \sum_{l=1}^{n_{sj}} \left[\boldsymbol{Z}_{sj,i_l} - \boldsymbol{Z}_{sj,m_{sj}+l}\right]$, and $\Delta_{sji} = X_{sji} - E(X_{sji}|\boldsymbol{W}_{sj}, \boldsymbol{Z}_{sj}, \sum_{i=1}^{m_{sj}+n_{sj}} Y_{sji} = n_{sj})$

B_{sj} is a subset of A'_{sj}, where $i \in \{i_1, i_2, \ldots, i_{n_{sj}}\}$, for $i = 1, 2, \ldots, m_{sj}$;

B'_{sj} is a subset of A'_{sj}, where $i, i' \in \{i_1, i_2, \ldots, i_{n_{sj}}\}$, for $1 \le i < i' \le m_{sj}$;

C_{sj} is a subset of A'_{sj}, where $i \notin \{i_1, i_2, \ldots, i_{n_{sj}}\}$, for $i = m_{sj}+1, m_{sj}+2, \ldots, m_{sj}+n_{sj}$

C'_{sj} is a subset of A'_{sj}, where $i, i' \notin \{i_1, i_2, \ldots, i_{n_{sj}}\}$, for $m_{sj}+1 \le i < i' \le m_{sj}+n_{sj}$

D_{sj} is a subset of B_{sj}, where $i' \notin \{i_1, i_2, \ldots, i_{n_{sj}}\}$, for $i' = m_{sj}+1, m_{sj}+2, \ldots, m_{sj}+n_{sj}$

Taking the expectation of L^*_{sj} conditional on $\boldsymbol{W}_{sj}, \boldsymbol{Z}_{sj}, \sum_{i=1}^{m_{sj}+n_{sj}} Y_{sji} = n_{sj}$ causes the first order term to disappear, and the second order term would be a function of $\boldsymbol{\beta}_X$ and the variance/covariance of $\boldsymbol{X}_{sj}$:

$$
\begin{aligned}
L_{sj} \approx\ & L^*_{sj}|_{\boldsymbol{X}_{sj}=\widetilde{\boldsymbol{X}}_{sj}} \\
& - \frac{1}{2}\sum_{i=1}^{m_{sj}} \frac{\begin{array}{c}\left[\sum_{(i_1,\ldots,i_{n_{sj}})\in B_{sj}} \exp\left(G_{i_l}\right)(\boldsymbol{\beta}_X^T \boldsymbol{f'}(X_{sji}))^2 + \sum_{(i_1,\ldots,i_{n_{sj}})\in B_{sj}} \exp(G_{i_l})\boldsymbol{\beta}_X^T \boldsymbol{f''}(X_{sji})\right] \\ \times (1+\sum_{(i_1,\ldots,i_{n_{sj}})\in A'_{sj}} \exp(G_{i_l})) - 2(\sum_{(i_1,\ldots,i_{n_{sj}})\in B_{sj}} \exp(G_{i_l})\boldsymbol{\beta}_X^T \boldsymbol{f'}(X_{sji}))^2\end{array}}{\left(1+\sum_{(i_1,\ldots,i_{n_{sj}})\in A'_{sj}} G_{i_l}\right)^3}|_{\boldsymbol{X}_{sj}=\widetilde{\boldsymbol{X}}_{sj}} \\
& \times Var\left(X_{sji}|\boldsymbol{W}_{sj}, \boldsymbol{Z}_{sj} \sum_{i=1}^{m_{sj}+n_{sj}} Y_{sji} = n_{sj}\right) \\
& - \frac{1}{2}\sum_{i=m_{sj}+1}^{m_{sj}+n_{sj}} \frac{\begin{array}{c}\left[\sum_{(i_1,\ldots,i_{n_{sj}})\in C_{sj}} \exp\left(G_{i_l}\right)(\boldsymbol{\beta}_X^T \boldsymbol{f'}(X_{sji}))^2 + \sum_{(i_1,\ldots,i_{n_{sj}})\in C_{sj}} \exp(G_{i_l})(-\boldsymbol{\beta}_X^T \boldsymbol{f''}(X_{sji}))\right] \\ \times (1+\sum_{(i_1,\ldots,i_{n_{sj}})\in A'_{sj}} \exp(G_{i_l})) - 2(\sum_{(i_1,\ldots,i_{n_{sj}})\in C_{sj}} \exp(G_{i_l})(-\boldsymbol{\beta}_X^T \boldsymbol{f'}(X_{sji})))^2\end{array}}{\left(1+\sum_{(i_1,\ldots,i_{n_{sj}})\in A'_{sj}} G_{i_l}\right)^3}|_{\boldsymbol{X}_{sj}=\widetilde{\boldsymbol{X}}_{sj}} \\
& \times Var\left(X_{sji}|\boldsymbol{W}_{sj}, \boldsymbol{Z}_{sj} \sum_{i=1}^{m_{sj}+n_{sj}} Y_{sji} = n_{sj}\right) \\
& - \sum_{1\le i<i'\le m_{sj}} \frac{\begin{array}{c}\left[\sum_{(i_1,\ldots,i_{n_{sj}})\in B'_{sj}} \exp(G_{i_l})(\boldsymbol{\beta}_X^T \boldsymbol{f'}(X_{sji}))(\boldsymbol{\beta}_X^T \boldsymbol{f'}(X_{sji'}))\right](1+\sum_{(i_1,\ldots,i_{n_{sj}})\in A'_{sj}} G_{i_l}) \\ -2\left(\sum_{(i_1,\ldots,i_{n_{sj}})\in B'_{sj}} \exp(G_{i_l})\boldsymbol{\beta}_x^T \boldsymbol{f'}(X_{sji})\right)\left(\sum_{(i_1,\ldots,i_{n_{sj}})\in B'_{sj}} \exp(G_{i_l})\boldsymbol{\beta}_x^T \boldsymbol{f'}(X_{sji'})\right)\end{array}}{(1+\sum_{(i_1,\ldots,i_{n_{sj}})\in A'_{sj}} G_{i_l})^3}|_{\boldsymbol{X}_{sj}=\widetilde{\boldsymbol{X}}_{sj}} \\
& \times Cov\left(X_{sji}, X_{sji'}|\boldsymbol{W}_{sj}, \boldsymbol{Z}_{sj}, \sum_{i=1}^{m_{sj}+n_{sj}} Y_{sji} = n_{sj}\right) \\
& - \sum_{m_{sj}+1\le i<i'\le m_{sj}+n_{sj}} \frac{\begin{array}{c}\left[\sum_{(i_1,\ldots,i_{n_{sj}})\in C'_{sj}} \exp(G_{i_l})(\boldsymbol{\beta}_X^T \boldsymbol{f'}(X_{sji}))(\boldsymbol{\beta}_X^T \boldsymbol{f'}(X_{sji'}))\right](1+\sum_{(i_1,\ldots,i_{n_{sj}})\in A'_{sj}} G_{i_l}) \\ -2\left(\sum_{(i_1,\ldots,i_{n_{sj}})\in C'_{sj}} \exp(G_{i_l})\boldsymbol{\beta}_x^T \boldsymbol{f'}(X_{sji})\right)\left(\sum_{(i_1,\ldots,i_{n_{sj}})\in C'_{sj}} \exp(G_{i_l})\boldsymbol{\beta}_x^T \boldsymbol{f'}(X_{sji'})\right)\end{array}}{(1+\sum_{(i_1,\ldots,i_{n_{sj}})\in A'_{sj}} G_{i_l})^3}|_{\boldsymbol{X}_{sj}=\widetilde{\boldsymbol{X}}_{sj}} \\
& \times Cov\left(X_{sji}, X_{sji'}|\boldsymbol{W}_{sj}, \boldsymbol{Z}_{sj}, \sum_{i=1}^{m_{sj}+n_{sj}} Y_{sji} = n_{sj}\right) \\
& - \sum_{1\le i\le m_{sj}<i'\le m_{sj}+n_{sj}} \frac{\begin{array}{c}\left[\sum_{(i_1,\ldots,i_{n_{sj}})\in D_{sj}} \exp(G_{i_l})(\boldsymbol{\beta}_X^T \boldsymbol{f'}(X_{sji}))(-\boldsymbol{\beta}_X^T \boldsymbol{f'}(X_{sji'}))\right](1+\sum_{(i_1,\ldots,i_{n_{sj}})\in A'_{sj}} G_{i_l}) \\ -2\left(\sum_{(i_1,\ldots,i_{n_{sj}})\in D_{sj}} \exp(G_{i_l})\boldsymbol{\beta}_x^T \boldsymbol{f'}(X_{sji})\right)\left(\sum_{(i_1,\ldots,i_{n_{sj}})\in D_{sj}} \exp(G_{i_l})(-\boldsymbol{\beta}_x^T \boldsymbol{f'}(X_{sji'}))\right)\end{array}}{(1+\sum_{(i_1,\ldots,i_{n_{sj}})\in A'_{sj}} G_{i_l})^3}|_{\boldsymbol{X}_{sj}=\widetilde{\boldsymbol{X}}_{sj}} \\
& \times Cov\left(X_{sji}, X_{sji'}|\boldsymbol{W}_{sj}, \boldsymbol{Z}_{sj}, \sum_{i=1}^{m_{sj}+n_{sj}} Y_{sji} = n_{sj}\right)
\end{aligned}
$$

If $\boldsymbol{\beta}_X$ or the variance/covariance terms are small, then the second order term will approaches 0, and therefore, the likelihood function from the jth stratum of sth study could be approximated by:

$$
\widetilde{L}_{sj} = \left(1 + \sum_{(i_1,\ldots,i_{n_{sj}})\in A'_{sj}} \exp\left(\boldsymbol{\beta}_X^T \sum_{l=1}^{n_{sj}} \left[\boldsymbol{f}(\widetilde{X}_{sj,i_l}) - \boldsymbol{f}(\widetilde{X}_{sj,m_{sj}+l})\right] + \boldsymbol{\beta}_{\mathbf{Z}}^T \sum_{l=1}^{n_{sj}} \left[\mathbf{Z}_{sj,i_l} - \mathbf{Z}_{sj,m_{sj}+l}\right]\right)\right)^{-1}
$$

where $\widetilde{X}_{sji} = E(X_{sji}|\boldsymbol{W}_{sj}, \boldsymbol{Z}_{sj}, \sum_{i=1}^{m_{sj}+n_{sj}} Y_{sji} = n_{sj})$

S2 Estimating Equations

In this section, we present the estimating equations.

Let $G_{i_l} = \boldsymbol{\beta}_X^T \sum_{l=1}^{n_{sj}} \left[\boldsymbol{f}(\widetilde{X}_{sji_l}) - \boldsymbol{f}(\widetilde{X}_{sj(m_{sj}+l)})\right] + \boldsymbol{\beta}_{\boldsymbol{Z}}^T \sum_{l=1}^{n_{sj}} \left[\boldsymbol{Z}_{sji_l} - \boldsymbol{Z}_{sj(m_{sj}+l)}\right]$. Let C_{sji} be an indicator function that takes on value q if the ith observation from the jth

stratum of the sth study is in the qth calibration subset, $q = 1, 2, \ldots, Q$. Note that for simplicity we usually arrange studies that needs calibration before studies that do not require calibration, such that $C_{sji} = s, s = 1, 2, \ldots, Q$ and $C_{sji} = 0, s > Q$. Suppose study s contains t_s strata;, then a total of $T = \sum_{s=1}^{S} t_s$ strata will contribute to the pooled analysis.

The set of estimating equations are:

$$\left[\boldsymbol{\psi_a}, \boldsymbol{\psi_b}, \boldsymbol{\psi_{\beta_X}}, \boldsymbol{\beta_{\beta_Z}}\right] = \mathbf{0}$$

and

$$\begin{aligned}\boldsymbol{\psi_a} &= \left(\sum_{s=1}^{S}\sum_{j=1}^{t_s}\sum_{i=1}^{m_{sj}} I(C_{sji} = 1)(X_{sji} - a_1 - b_1 W_{sji}), \ldots, \sum_{s=1}^{S}\sum_{j=1}^{t_s}\sum_{i=1}^{m_{sj}} I(C_{sji} = Q)(X_{sji} - a_1 - b_1 W_{sji})\right) \\ &= \left(\sum_{s=1}^{S}\sum_{j=1}^{t_s} \psi_{a_1}^{(sj)}, \ldots, \sum_{s=1}^{S}\sum_{j=1}^{t_s} \psi_{a_Q}^{(sj)}\right),\end{aligned}$$

$$\begin{aligned}\boldsymbol{\psi_b} =& \Bigg(\sum_{s=1}^{S}\sum_{j=1}^{t_s}\sum_{i=1}^{m_{sj}} I(C_{sji} = 1)(X_{sji} - a_1 - b_1 W_{sji}) W_{sji}, \ldots, \\ & \sum_{s=1}^{S}\sum_{j=1}^{t_s}\sum_{i=1}^{m_{sj}} I(C_{sji} = Q)(X_{sji} - a_1 - b_1 W_{sji}) W_{sji}\Bigg) \\ =& \left(\sum_{s=1}^{S}\sum_{j=1}^{t_s} {}_{b_1}^{(sj)}, \ldots, \sum_{s=1}^{S}\sum_{j=1}^{t_s} {}_{b_Q}^{(sj)}\right),\end{aligned}$$

$$\begin{aligned}\boldsymbol{\psi_{\beta_X}} &= \left(\sum_{s=1}^{S}\sum_{j=1}^{t_s} \frac{\partial}{\partial \beta_{X_1}} \ell_{sj}, \ldots, \sum_{s=1}^{S}\sum_{j=1}^{t_s} \frac{\partial}{\partial \beta_{X_K}} \ell_{sj}\right) \\ &= \left(\sum_{s=1}^{S}\sum_{j=1}^{t_s} {}_{\beta_{X_1}}^{(sj)}, \ldots, \sum_{s=1}^{S}\sum_{j=1}^{t_s} {}_{\beta_{X_K}}^{(sj)}\right),\end{aligned}$$

$$\begin{aligned}\boldsymbol{\psi_{\beta_Z}} &= \left(\sum_{s=1}^{S}\sum_{j=1}^{t_s} \frac{\partial}{\partial \beta_{Z_1}} \ell_{sj}, \ldots, \sum_{s=1}^{S}\sum_{j=1}^{t_s} \frac{\partial}{\partial \beta_{Z_P}} \ell_{sj}\right) \\ &= \left(\sum_{s=1}^{S}\sum_{j=1}^{t_s} {}_{\beta_{Z_1}}^{(sj)}, \ldots, \sum_{s=1}^{S}\sum_{j=1}^{t_s} {}_{\beta_{Z_P}}^{(sj)}\right),\end{aligned}$$

with

$$
\begin{aligned}
\widetilde{\ell}_{sj} =& \log\left(\widetilde{L}_{sj}\right) \\
=& -\log\left(1 + \sum_{(i_1,\ldots,i_{n_{sj}})\in A'_{sj}} \exp\left(\boldsymbol{\beta}_X^T \sum_{l=1}^{n_{sj}} \left[\boldsymbol{f}(\widetilde{X}_{sj,i_l}) - \boldsymbol{f}(\widetilde{X}_{sj,m_{sj}+l})\right] + \boldsymbol{\beta}_{\boldsymbol{Z}} \sum_{l=1}^{n_{sj}} \left[\boldsymbol{Z}_{sj,i_l} - \boldsymbol{Z}_{\boldsymbol{sj},\boldsymbol{m_{sj}}+\boldsymbol{l}}\right]\right)\right) \\
{}^{(sj)}_{\beta_{X_k}} =& \frac{\partial}{\partial \beta_{X_k}} \ell_{sj} = -\frac{\sum_{(i_1,\ldots,i_{n_{sj}})\in A'_{sj}} \exp(G_{i_l}) \left(\sum_{l=1}^{n_{sj}} \left[f_k(\widetilde{X}_{sj,i_l}) - f_k(\widetilde{X}_{sj,m_{sj}+l})\right]\right)}{1 + \sum_{(i_1,\ldots,i_{n_{sj}})\in A'_{sj}} \exp(G_{i_l})}, k = 1, 2, \ldots, K \\
{}^{(sj)}_{\beta_{Z_p}} =& \frac{\partial}{\partial \beta_{Z_p}} \ell_{sj} = -\frac{\sum_{(i_1,\ldots,i_{n_{sj}})\in A'_{sj}} \exp(G_{i_l}) \left(\sum_{l=1}^{n_{sj}} \left[Z_{p,sj,i_l} - Z_{p,sj,m_{sj}+l})\right]\right)}{1 + \sum_{(i_1,\ldots,i_{n_{sj}})\in A'_{sj}} \exp(G_{i_l})}, p = 1, 2, \ldots, P
\end{aligned}
\tag{2}
$$

Here $f_k(\widetilde{X}_{sji})$ is the kth component of $\boldsymbol{f}(\widetilde{X}_{sji})$, and $Z_{p,sji}$ is the pth component of $\boldsymbol{Z}_{sji}$

S3 Sandwich Variance Estimator

The parameters to be estimated are $\boldsymbol{\beta} = [\boldsymbol{\beta}_X, \boldsymbol{\beta}_{\boldsymbol{Z}}]$, $\boldsymbol{a} = [a_1, a_2, \ldots, a_Q]$, $\boldsymbol{b} = [b_1, b_2, \ldots, b_Q]$, which are collectively denoted as: $\boldsymbol{\theta} = [\boldsymbol{a}, \boldsymbol{b}, \boldsymbol{\beta}]$. The sandwich variance estimator is: $\widehat{V}(\boldsymbol{\theta}) = \widehat{B}(\boldsymbol{\theta})^{-1}\widehat{A}(\boldsymbol{\theta})(\widehat{B}(\boldsymbol{\theta})^{-1})^T$ where $\widehat{A}(\boldsymbol{\theta})$ and $\widehat{B}(\boldsymbol{\theta})$ are $2Q + K + P$ dimensional square matrices.

Let $\boldsymbol{\psi}_{\boldsymbol{\theta}_{sj}} = \left[\ {}^{(sj)}_{a_1}, \ldots, \ {}^{(sj)}_{a_Q}, \ {}^{(sj)}_{b_1}, \ldots, \psi^{(sj)}_{b_Q}, \psi^{(sj)}_{\beta_{X_1}}, \ldots, \ {}^{(sj)}_{\beta_{X_K}}, \ {}^{(sj)}_{\beta_{Z_1}}, \ldots, \ {}^{(sj)}_{\beta_{Z_P}}\right]^T$. The matrix components of $\widehat{A}(\boldsymbol{\theta})$ and $\widehat{B}(\boldsymbol{\theta})$ are:

$$
\begin{aligned}
\widehat{A}(\boldsymbol{\theta}) =& \sum_{s=1}^{S} \sum_{j}^{t_s} \boldsymbol{\psi}_{\boldsymbol{\theta}_{sj}} \boldsymbol{\psi}_{\boldsymbol{\theta}_{sj}}^T , \\
\widehat{B}(\boldsymbol{\theta}) =& \sum_{s=1}^{S} \sum_{j}^{t_s} \frac{\partial \boldsymbol{\psi}_{\boldsymbol{\theta}_{sj}}}{\partial \boldsymbol{\theta}^T} .
\end{aligned}
$$

The derivatives of $\boldsymbol{\psi}_{\boldsymbol{\theta}_{sj}}$ are:

$$
\begin{aligned}
\frac{\partial \psi_{a_q}^{(sj)}}{\partial a_q} &= -\sum_{i=1}^{m_{sj}} I(C_{sji} = q), \quad q = 1, 2, \ldots, Q \\
\frac{\partial \psi_{a_q}^{(sj)}}{\partial a_{q'}} &= 0, \quad q \neq q' \in (1, 2, \ldots, Q) \\
\frac{\partial \psi_{a_q}^{(sj)}}{\partial b_q} &= -\sum_{i=1}^{m_{sj}} I(C_{sji} = q) W_{sji}, \quad q = 1, 2, \ldots, Q \\
\frac{\partial \psi_{a_q}^{(sj)}}{\partial b_{q'}} &= 0, \quad q \neq q' \in (1, 2, \ldots, Q) \\
\frac{\partial \psi_{a_q}^{(sj)}}{\partial \beta_{X_k}} &= 0, \quad q = 1, 2, \ldots, Q; \quad k = 1, 2, \ldots K \\
\frac{\partial \psi_{a_q}^{(sj)}}{\partial \beta_{Z_p}} &= 0, \quad q = 1, 2, \ldots, Q; \quad p = 1, 2, \ldots P
\end{aligned}
$$

$$
\begin{aligned}
\frac{\partial \psi_{b_q}^{(sj)}}{\partial a_q} &= -\sum_{i=1}^{m_{sj}} I(C_{sji} = q) W_{sji}, \quad q = 1, 2, \ldots, Q \\
\frac{\partial \psi_{b_q}^{(sj)}}{\partial a_{q'}} &= 0, \quad q \neq q' \in (1, 2, \ldots, Q) \\
\frac{\partial \psi_{b_q}^{(sj)}}{\partial b_q} &= -\sum_{i=1}^{m_{sj}} I(C_{sji} = q) W_{sji}^2, \quad q = 1, 2, \ldots, Q \\
\frac{\partial \psi_{b_q}^{(sj)}}{\partial b_{q'}} &= 0, \quad q \neq q' \in (1, 2, \ldots, Q) \\
\frac{\partial \psi_{b_q}^{(sj)}}{\partial \beta_{X_k}} &= 0, \quad q = 1, 2, \ldots, Q; \quad k = 1, 2, \ldots K \\
\frac{\partial \psi_{b_q}^{(sj)}}{\partial \beta_{Z_p}} &= 0, \quad q = 1, 2, \ldots, Q; \quad p = 1, 2, \ldots P
\end{aligned}
$$

For the full calibration method, if the sth $(s = 1, 2, \ldots, S)$ study is not the qth $(q = 1, 2, \ldots, Q)$ study that needs calibration, we have

$$
\begin{aligned}
\frac{\partial \psi_{\beta_{X_k}^{(sj)}}}{\partial a_q} &= 0 \\
\frac{\partial \psi_{\beta_{X_k}^{(sj)}}}{\partial b_q} &= 0
\end{aligned}
$$

where $k = 1, 2, \ldots, K$

However, if the sth $(s = 1, 2, \ldots, S)$ study is the qth $(q = 1, 2, \ldots, Q)$ study that needs calibration, we have

$$
\begin{aligned}
\frac{\partial \psi_{\beta_{X_k}^{(sj)}}}{\partial a_q} = \Bigg\{ \Bigg[& \sum_{(i_1, \ldots, i_{n_{sj}}) \in A'_{sj}} \exp\left(G_{i_l}\right) \left(\boldsymbol{\beta}_X \sum_{l=1}^{n_{sj}} \left[\boldsymbol{f'}(\widetilde{X}_{sj,i_l}) - \boldsymbol{f'}(\widetilde{X}_{sj,m_{sj}+l}) \right] \right) \left(\sum_{l=1}^{n_{sj}} \left[f_k(\widetilde{X}_{sj,i_l}) - f_k(\widetilde{X}_{sj,m_{sj}+l}) \right] \right) \\
& + \sum_{(i_1, \ldots, i_{n_{sj}}) \in A'_{sj}} \exp(G_{i_l}) \left(\sum_{l=1}^{n_{sj}} \left[\boldsymbol{f'}(\widetilde{X}_{sj,i_l}) - \boldsymbol{f'}(\widetilde{X}_{sj,m_{sj}+l}) \right] \right) \Bigg] \left(1 + \sum_{(i_1, \ldots, i_{n_{sj}}) \in A'_{sj}} \exp(G_{i_l}) \right) \\
& - \left(\sum_{(i_1, \ldots, i_{n_{sj}}) \in A'_{sj}} \exp\left(G_{i_l}\right) \left(\sum_{l=1}^{n_{sj}} \left[f_k(\widetilde{X}_{sj,i_l}) - f_k(\widetilde{X}_{sj,m_{sj}+l}) \right] \right) \right) \\
& \times \left(\sum_{(i_1, \ldots, i_{n_{sj}}) \in A'_{sj}} \exp\left(G_{i_l}\right) \left(\boldsymbol{\beta}_X^T \sum_{l=1}^{n_{sj}} \left[\boldsymbol{f'}(\widetilde{X}_{sj,i_l}) - \boldsymbol{f'}(\widetilde{X}_{sj,m_{sj}+l}) \right] \right) \right) \Bigg\} \Bigg/ \Bigg\{ - \left(1 + \sum_{(i_1, \ldots, i_{n_{sj}}) \in A'_{sj}} \exp(G_{i_l}) \right)^2 \Bigg\}
\end{aligned}
$$

where β_{X_k} is the kth component of $\boldsymbol{\beta}_X$ $(k = 1, 2, \ldots, K)$, and a_q $(q = 1, 2, \ldots, Q)$ is the intercept of the qth calibration study

$$
\begin{aligned}
\frac{\partial\psi_{\beta^{(sj)}_{X_k}}}{\partial b_q} = &\Bigg\{\Bigg[\sum_{(i_1,\ldots,i_{n_{sj}})\in A'_{sj}} \exp\left(G_{i_l}\right)\left(\boldsymbol{\beta}_X^T\sum_{l=1}^{n_{sj}}\left[\boldsymbol{f'}(\widetilde{X}_{sj,i_l})W_{sj,i_l} - \boldsymbol{f'}(\widetilde{X}_{sj,m_{sj}+l})W_{sj,m_{sj}+l}\right]\right)\times\left(\sum_{l=1}^{n_{sj}}\left[f_k(\widetilde{X}_{sj,i_l}) - f_k(\widetilde{X}_{sj,m_{sj}+l})\right]\right)\\
&+\sum_{(i_1,\ldots,i_{n_{sj}})\in A'_{sj}}\exp(G_{i_l})\left(\sum_{l=1}^{n_{sj}}\left[\boldsymbol{f'}(\widetilde{X}_{sj,i_l})W_{sj,i_l} - \boldsymbol{f'}(\widetilde{X}_{sj,m_{sj}+l})W_{sj,m_{sj}+l}\right]\right)\Bigg]\left(1+\sum_{(i_1,\ldots,i_{n_{sj}})\in A'_{sj}}\exp(G_{i_l})\right)\\
&-\left(\sum_{(i_1,\ldots,i_{n_{sj}})\in A'_{sj}}\exp\left(G_{i_l}\right)\left(\sum_{l=1}^{n_{sj}}\left[f_k(\widetilde{X}_{sj,i_l}) - f_k(\widetilde{X}_{sj,m_{sj}+l})\right]\right)\right)\\
&\times\left(\sum_{(i_1,\ldots,i_{n_{sj}})\in A'_{sj}}\exp\left(G_{i_l}\right)\left(\boldsymbol{\beta}_X^T\sum_{l=1}^{n_{sj}}\left[\boldsymbol{f'}(\widetilde{X}_{sj,i_l})W_{sj,i_l} - \boldsymbol{f'}(\widetilde{X}_{sj,m_{sj}+l})W_{sj,m_{sj}+l}\right]\right)\right)\Bigg]\Bigg\}\\
&\Bigg/\left\{-\left(1+\sum_{(i_1,\ldots,i_{n_{sj}})\in A'_{sj}}\exp(G_{i_l})\right)^2\right\}
\end{aligned}
$$

For the internalized calibration method, let H_{sji} be an indicator variable taking on value 1 if the ith observation from the jth stratum of sth study is used in calibration.

$$
\begin{aligned}
\frac{\partial\psi_{\beta^{(sj)}_{X_k}}}{\partial a_q} = &\Bigg\{\Bigg[\sum_{(i_1,\ldots,i_{n_{sj}})\in A'_{sj}} \exp\left(G_{i_l}\right)\left(\boldsymbol{\beta}_X\sum_{l=1}^{n_{sj}}\left[\boldsymbol{f'}(\widetilde{X}_{sj,i_l})I(H_{sj,i_l}=0) - \boldsymbol{f'}(\widetilde{X}_{sj,m_{sj}+l})\right]\right)\times\left(\sum_{l=1}^{n_{sj}}\left[f_k(\widetilde{X}_{sj,i_l}) - f_k(\widetilde{X}_{sj,m_{sj}+l})\right]\right)\\
&+\sum_{(i_1,\ldots,i_{n_{sj}})\in A'_{sj}}\exp(G_{i_l})\left(\sum_{l=1}^{n_{sj}}\left[\boldsymbol{f'}(\widetilde{X}_{sj,i_l})I(H_{sj,i_l}=0) - \boldsymbol{f'}(\widetilde{X}_{sj,m_{sj}+l})\right]\right)\Bigg]\left(1+\sum_{(i_1,\ldots,i_{n_{sj}})\in A'_{sj}}\exp(G_{i_l})\right)\\
&-\left(\sum_{(i_1,\ldots,i_{n_{sj}})\in A'_{sj}}\exp\left(G_{i_l}\right)\left(\sum_{l=1}^{n_{sj}}\left[f_k(\widetilde{X}_{sj,i_l}) - f_k(\widetilde{X}_{sj,m_{sj}+l})\right]\right)\right)\\
&\times\left(\sum_{(i_1,\ldots,i_{n_{sj}})\in A'_{sj}}\exp\left(G_{i_l}\right)\left(\boldsymbol{\beta}_X^T\sum_{l=1}^{n_{sj}}\left[\boldsymbol{f'}(\widetilde{X}_{sj,i_l})I(H_{sji_l}=0) - \boldsymbol{f'}(\widetilde{X}_{sj,m_{sj}+l})\right]\right)\right)\Bigg\}\Bigg/\left\{-\left(1+\sum_{A'_{sj}}\exp(G_{i_l})\right)^2\right\}
\end{aligned}
$$

$$
\begin{aligned}
\frac{\partial\psi_{\beta^{(sj)}_{X_k}}}{\partial b_q} = &\Bigg\{\Bigg[\sum_{(i_1,\ldots,i_{n_{sj}})\in A'_{sj}} \exp\left(G_{i_l}\right)\left(\boldsymbol{\beta}_X^T\sum_{l=1}^{n_{sj}}\left[\boldsymbol{f'}(\widetilde{X}_{sj,i_l})W_{sj,i_l}I(H_{sj,i_l}=0) - \boldsymbol{f'}(\widetilde{X}_{sj,m_{sj}+l})W_{sj,m_{sj}+l}\right]\right)\\
&\times\left(\sum_{l=1}^{n_{sj}}\left[f_k(\widetilde{X}_{sj,i_l}) - f_k(\widetilde{X}_{sj,m_{sj}+l})\right]\right)\\
&+\sum_{(i_1,\ldots,i_{n_{sj}})\in A'_{sj}}\exp(G_{i_l})\left(\sum_{l=1}^{n_{sj}}\left[\boldsymbol{f'}(\widetilde{X}_{sj,i_l})W_{sj,i_l}I(H_{sj,i_l}=0) - \boldsymbol{f'}(\widetilde{X}_{sj,m_{sj}+l})W_{sj,m_{sj}+l}\right]\right)\Bigg]\times\left(1+\sum_{(i_1,\ldots,i_{n_{sj}})\in A'_{sj}}\exp(G_{i_l})\right)\\
&-\left(\sum_{(i_1,\ldots,i_{n_{sj}})\in A'_{sj}}\exp\left(G_{i_l}\right)\left(\sum_{l=1}^{n_{sj}}\left[f_k(\widetilde{X}_{sj,i_l}) - f_k(\widetilde{X}_{sj,m_{sj}+l})\right]\right)\right)\\
&\times\left(\sum_{(i_1,\ldots,i_{n_{sj}})\in A'_{sj}}\exp\left(G_{i_l}\right)\left(\boldsymbol{\beta}_X^T\sum_{l=1}^{n_{sj}}\left[\boldsymbol{f'}(\widetilde{X}_{sj,i_l})W_{sj,i_l}I(H_{sj,i_l}=0) - \boldsymbol{f'}(\widetilde{X}_{sj,m_{sj}+l})W_{sj,m_{sj}+l}\right]\right)\right)\Bigg\}\\
&\Bigg/\left\{-\left(1+\sum_{(i_1,\ldots,i_{n_{sj}})\in A'_{sj}}\exp(G_{i_l})\right)^2\right\}
\end{aligned}
$$

$$\frac{\partial \psi^{(sj)}_{\beta_{X_k}}}{\partial \beta_{X_k}} = \Bigg\{ \sum_{(i_1,\ldots,i_{n_{sj}})\in A'_{sj}} \exp(G_{i_l}) \Bigg(\sum_{l=1}^{n_{sj}} \Big[f_k(\widetilde{X}_{sj,i_l}) - f_k(\widetilde{X}_{sj,m_{sj}+l}) \Big] \Bigg)^2 \Bigg(1 + \sum_{(i_1,\ldots,i_{n_{sj}})\in A'_{sj}} \exp(G_{i_l}) \Bigg)$$
$$- \Bigg(\sum_{(i_1,\ldots,i_{n_{sj}})\in A'_{sj}} \exp(G_{i_l}) \Bigg(\sum_{l=1}^{n_{sj}} \Big[f_k(\widetilde{X}_{sj,i_l}) - f_k(\widetilde{X}_{sj,m_{sj}+l}) \Big] \Bigg) \Bigg)^2 \Bigg\} \Bigg/ \Bigg\{ -(1 + \sum_{(i_1,\ldots,i_{n_{sj}})\in A'_{sj}} \exp(G_{i_l}))^2 \Bigg\}$$

$$\frac{\partial \psi^{(sj)}_{\beta_{X_k}}}{\partial \beta_{X_{k'}}} = \Bigg\{ \sum_{(i_1,\ldots,i_{n_{sj}})\in A'_{sj}} \exp(G_{i_l}) \Bigg(\sum_{l=1}^{n_{sj}} \Big[f_k(\widetilde{X}_{sj,i_l}) - f_k(\widetilde{X}_{sj,m_{sj}+l}) \Big] \Bigg)$$
$$\times \Bigg(\sum_{l=1}^{n_{sj}} \Big[f_{k'}(\widetilde{X}_{sj,i_l}) - f_{k'}(\widetilde{X}_{sj,m_{sj}+l}) \Big] \Bigg) \Bigg(1 + \sum_{(i_1,\ldots,i_{n_{sj}})\in A'_{sj}} \exp(G_{i_l}) \Bigg)$$
$$- \Bigg(\sum_{(i_1,\ldots,i_{n_{sj}})\in A'_{sj}} \exp(G_{i_l}) \Bigg(\sum_{l=1}^{n_{sj}} \Big[f_k(\widetilde{X}_{sj,i_l}) - f_k(\widetilde{X}_{sj,m_{sj}+l}) \Big] \Bigg) \Bigg)$$
$$\times \Bigg(\sum_{(i_1,\ldots,i_{n_{sj}})\in A'_{sj}} \exp(G_{i_l}) \Bigg(\sum_{l=1}^{n_{sj}} \Big[f_{k'}(\widetilde{X}_{sj,i_l}) - f_{k'}(\widetilde{X}_{sj,m_{sj}+l}) \Big] \Bigg) \Bigg) \Bigg\} \Bigg/ \Bigg\{ -(1 + \sum_{(i_1,\ldots,i_{n_{sj}})\in A'_{sj}} \exp(G_{i_l}))^2 \Bigg\}$$

where $k \neq k' \in (1, 2, \ldots, K)$

$$\frac{\partial \psi^{(sj)}_{\beta_{X_k}}}{\partial \beta_{Z_p}} = \Bigg\{ \sum_{(i_1,\ldots,i_{n_{sj}})\in A'_{sj}} \exp(G_{i_l}) \Bigg(\sum_{l=1}^{n_{sj}} \Big[f_k(\widetilde{X}_{sj,i_l}) - f_k(\widetilde{X}_{sj,m_{sj}+l}) \Big] \Bigg)$$
$$\times \Bigg(\sum_{l=1}^{n_{sj}} \big[Z_{p,sj,i_l} - Z_{p,sj,m_{sj}+l} \big] \Bigg) \Bigg(1 + \sum_{(i_1,\ldots,i_{n_{sj}})\in A'_{sj}} \exp(G_{i_l}) \Bigg)$$
$$- \Bigg(\sum_{(i_1,\ldots,i_{n_{sj}})\in A'_{sj}} \exp(G_{i_l}) \Bigg(\sum_{l=1}^{n_{sj}} \Big[f_k(\widetilde{X}_{sj,i_l}) - f_k(\widetilde{X}_{sj,m_{sj}+l}) \Big] \Bigg) \Bigg)$$
$$\times \Bigg(\sum_{(i_1,\ldots,i_{n_{sj}})\in A'_{sj}} \exp(G_{i_l}) \Bigg(\sum_{l=1}^{n_{sj}} \big[Z_{p,sj,i_l} - Z_{p,sj,m_{sj}+l} \big] \Bigg) \Bigg) \Bigg\} \Bigg/ \Bigg\{ -(1 + \sum_{(i_1,\ldots,i_{n_{sj}})\in A'_{sj}} \exp(G_{i_l}))^2 \Bigg\}$$

where β_{Z_p} is the pth $(p = 1, 2, \ldots, P)$ component of $\boldsymbol{\beta_Z}$.

For the full calibration method, if the sth $(s = 1, 2, \ldots, S)$ study is not the qth $(q = 1, 2, \ldots, Q)$ study that needs calibration, we have

$$\frac{\partial \psi_{\beta^{(sj)}_{Z_p}}}{\partial a_q} = 0$$
$$\frac{\partial \psi_{\beta^{(sj)}_{Z_p}}}{\partial b_q} = 0$$

where $p = 1, 2, \ldots, P$

However, if the sth $(s = 1, 2, \ldots, S)$ study is the qth $(q = 1, 2, \ldots, Q)$ study that needs calibration, we have

$$\frac{\partial \psi_{\beta_{Z_p}^{(sj)}}}{\partial a_q} = \Bigg\{ \Bigg[\sum_{(i_1,\ldots,i_{n_{sj}}) \in A'_{sj}} \exp\left(G_{i_l}\right) \left(\boldsymbol{\beta}_X \sum_{l=1}^{n_{sj}} \left[\boldsymbol{f'}(\widetilde{X}_{sj,i_l}) - \boldsymbol{f'}(\widetilde{X}_{sj,m_{sj}+l}) \right] \right) \times \left(\sum_{l=1}^{n_{sj}} \left[Z_{p,sj,i_l} - Z_{p,sj,m_{sj}+l} \right] \right) \Bigg] \left(1 + \sum_{(i_1,\ldots,i_{n_{sj}}) \in A'_{sj}} \exp(G_{i_l}) \right) - \left(\sum_{(i_1,\ldots,i_{n_{sj}}) \in A'_{sj}} \exp\left(G_{i_l}\right) \left(\sum_{l=1}^{n_{sj}} \left[Z_{p,sj,i_l} - Z_{p,sj,m_{sj}+l} \right] \right) \right) \times \left(\sum_{(i_1,\ldots,i_{n_{sj}}) \in A'_{sj}} \exp\left(G_{i_l}\right) \left(\boldsymbol{\beta}_X^T \sum_{l=1}^{n_{sj}} \left[\boldsymbol{f'}(\widetilde{X}_{sj,i_l}) - \boldsymbol{f'}(\widetilde{X}_{sj,m_{sj}+l}) \right] \right) \right) \Bigg\} \Bigg/ \Bigg\{ - \left(1 + \sum_{(i_1,\ldots,i_{n_{sj}}) \in A'_{sj}} \exp(G_{i_l}) \right)^2 \Bigg\}$$

$$\frac{\partial \psi_{\beta_{Z_p}^{(sj)}}}{\partial b_q} = \Bigg\{ \Bigg[\sum_{(i_1,\ldots,i_{n_{sj}}) \in A'_{sj}} \exp\left(G_{i_l}\right) \left(\boldsymbol{\beta}_X \sum_{l=1}^{n_{sj}} \left[\boldsymbol{f'}(\widetilde{X}_{sj,i_l}) W_{sj,i_l} - \boldsymbol{f'}(\widetilde{X}_{sj,m_{sj}+l}) W_{sj,m_{sj}+l} \right] \right) \times \left(\sum_{l=1}^{n_{sj}} \left[Z_{p,sj,i_l} - Z_{p,sj,m_{sj}+l} \right] \right) \Bigg] \left(1 + \sum_{(i_1,\ldots,i_{n_{sj}}) \in A'_{sj}} \exp(G_{i_l}) \right) - \left(\sum_{(i_1,\ldots,i_{n_{sj}}) \in A'_{sj}} \exp\left(G_{i_l}\right) \left(\sum_{l=1}^{n_{sj}} \left[Z_{p,sj,i_l} - Z_{p,sj,m_{sj}+l} \right] \right) \right) \times \left(\sum_{(i_1,\ldots,i_{n_{sj}}) \in A'_{sj}} \exp\left(G_{i_l}\right) \left(\boldsymbol{\beta}_X^T \sum_{l=1}^{n_{sj}} \left[\boldsymbol{f'}(\widetilde{X}_{sj,i_l}) W_{sj,i_l} - \boldsymbol{f'}(\widetilde{X}_{sj,m_{sj}+l}) W_{sj,m_{sj}+l} \right] \right) \right) \Bigg\} \Bigg/ \Bigg\{ - \left(1 + \sum_{(i_1,\ldots,i_{n_{sj}}) \in A'_{sj}} \exp(G_{i_l}) \right)^2 \Bigg\}$$

For internalized calibration method, we have

$$\frac{\partial \psi_{\beta_{Z_p}^{(sj)}}}{\partial a_q} = \Bigg\{ \Bigg\{ \Bigg[\sum_{(i_1,\ldots,i_{n_{sj}}) \in A'_{sj}} \exp\left(G_{i_l}\right) \left(\boldsymbol{\beta}_X \sum_{l=1}^{n_{sj}} \left[\boldsymbol{f'}(\widetilde{X}_{sj,i_l}) I(H_{sj,i_l} = 0) - \boldsymbol{f'}(\widetilde{X}_{sj,m_{sj}+l}) \right] \right) \times \left(\sum_{l=1}^{n_{sj}} \left[Z_{p,sj,i_l} - Z_{p,sj,m_{sj}+l} \right] \right) \Bigg] \left(1 + \sum_{(i_1,\ldots,i_{n_{sj}}) \in A'_{sj}} \exp(G_{i_l}) \right) - \left(\sum_{(i_1,\ldots,i_{n_{sj}}) \in A'_{sj}} \exp\left(G_{i_l}\right) \left(\sum_{l=1}^{n_{sj}} \left[Z_{p,sj,i_l} - Z_{p,sj,m_{sj}+l} \right] \right) \right) \times \left(\sum_{(i_1,\ldots,i_{n_{sj}}) \in A'_{sj}} \exp\left(G_{i_l}\right) \left(\boldsymbol{\beta}_X^T \sum_{l=1}^{n_{sj}} \left[\boldsymbol{f'}(\widetilde{X}_{sj,i_l}) I(H_{sj,i_l} = 0) - \boldsymbol{f'}(\widetilde{X}_{sj,m_{sj}+l}) \right] \right) \right) \Bigg\} \Bigg/ \Bigg\{ - \left(1 + \sum_{(i_1,\ldots,i_{n_{sj}}) \in A'_{sj}} \exp(G_{i_l}) \right)^2 \Bigg\}$$

$$\frac{\partial \psi_{\beta_{Z_p}^{(sj)}}}{\partial b_q} = \Bigg\{ \Bigg[\sum_{(i_1,\ldots,i_{n_{sj}})\in A'_{sj}} \exp\left(G_{i_l}\right) \left(\boldsymbol{\beta}_X \sum_{l=1}^{n_{sj}} \left[\boldsymbol{f'}(\widetilde{X}_{sj,i_l}) W_{sj,i_l} I(H_{sj,i_l}=0) - \boldsymbol{f'}(\widetilde{X}_{sj,m_{sj}+l}) W_{sj,m_{sj}+l} \right] \right)$$

$$\times \left(\sum_{l=1}^{n_{sj}} \left[Z_{p,sj,i_l} - Z_{p,sj,m_{sj}+l} \right] \right) \Bigg] \left(1 + \sum_{(i_1,\ldots,i_{n_{sj}})\in A'_{sj}} \exp(G_{i_l}) \right)$$

$$- \left(\sum_{(i_1,\ldots,i_{n_{sj}})\in A'_{sj}} \exp\left(G_{i_l}\right) \left(\sum_{l=1}^{n_{sj}} \left[Z_{p,sj,i_l} - Z_{p,sj,m_{sj}+l} \right] \right) \right)$$

$$\times] \left(\sum_{(i_1,\ldots,i_{n_{sj}})\in A'_{sj}} \exp\left(G_{i_l}\right) \left(\boldsymbol{\beta}_X^T \sum_{l=1}^{n_{sj}} \left[\boldsymbol{f'}(\widetilde{X}_{sj,i_l}) W_{sj,i_l} I(H_{sj,i_l}=0) - \boldsymbol{f'}(\widetilde{X}_{sj,m_{sj}+l}) W_{sj,m_{sj}+l} \right] \right) \right) \Bigg\}$$

$$\Bigg/ \left\{ -\left(1 + \sum_{(i_1,\ldots,i_{n_{sj}})\in A'_{sj}} \exp(G_{i_l}) \right)^2 \right\}$$

$$\frac{\partial \psi_{Z_p}^{(sj)}}{\partial \beta_{Z_p}} = \Bigg\{ \left(\sum_{(i_1,\ldots,i_{n_{sj}})\in A'_{sj}} \exp(G_{i_l}) \left(\sum_{l=1}^{n_{sj}} \left[Z_{p,sj,i_l} - Z_{p,sj,m_{sj}+l} \right] \right)^2 \right) \left(1 + \sum_{(i_1,\ldots,i_{n_{sj}})\in A'_{sj}} \exp(G_{i_l}) \right)$$

$$- \left(\sum_{(i_1,\ldots,i_{n_{sj}})\in A'_{sj}} \exp(G_{i_l}) \left(\sum_{l=1}^{n_{sj}} \left[Z_{p,sj,i_l} - Z_{p,sj,m_{sj}+l} \right] \right) \right)^2 \Bigg\} \Bigg/ \left\{ -\left(1 + \sum_{(i_1,\ldots,i_{n_{sj}})\in A'_{sj}} \exp(G_{i_l}) \right)^2 \right\}$$

$$\frac{\partial \psi_{Z_p}^{(sj)}}{\partial \beta_{Z'_p}} = \Bigg\{ \left(\sum_{(i_1,\ldots,i_{n_{sj}})\in A'_{sj}} \exp(G_{i_l}) \left(\sum_{l=1}^{n_{sj}} \left[Z_{p,sj,i_l} - Z_{p,sj,m_{sj}+l} \right] \right) \left(\sum_{l=1}^{n_{sj}} \left[Z_{p',sj,i_l} - Z_{p',sj,m_{sj}+l} \right] \right) \right) \left(1 + \sum_{(i_1,\ldots,i_{n_{sj}})\in A'_{sj}} \exp(G_{i_l}) \right)$$

$$- \left(\sum_{(i_1,\ldots,i_{n_{sj}})\in A'_{sj}} \exp(G_{i_l}) \left(\sum_{l=1}^{n_{sj}} \left[Z_{p,sj,i_l} - Z_{p,sj,m_{sj}+l} \right] \right) \right) \times \left(\sum_{(i_1,\ldots,i_{n_{sj}})\in A'_{sj}} \exp(G_{i_l}) \left(\sum_{l=1}^{n_{sj}} \left[Z_{p',sj,i_l} - Z_{p',sj,m_{sj}+l} \right] \right) \right) \Bigg\}$$

$$\Bigg/ \left\{ -\left(1 + \sum_{(i_1,\ldots,i_{n_{sj}})\in A'_{sj}} \exp(G_{i_l}) \right)^2 \right\}$$

where $k \neq k' \in (1, 2, \ldots, K)$

S4 Comparing Internalized and Full Calibration Method

The simulation results showed that full calibration method has more robust performance than internalized calibration method especially when the calibration proportion is large. In this section, we will give a mathematical justification for this phenomenon.

Recall that the approximate likelihood contributed from the jth stratum of sth study is:

$$\widetilde{L}_{sj} = \left(1 + \sum_{(i_1,\ldots,i_{n_{sj}})\in A'_{sj}} \exp\left(\boldsymbol{\beta}_X^T \sum_{l=1}^{n_{sj}} \left[\boldsymbol{f}(\widetilde{X}_{sj,i_l}) - \boldsymbol{f}(\widetilde{X}_{sj,m_{sj}+l}) \right] + \boldsymbol{\beta}_{\boldsymbol{Z}}^T \sum_{l=1}^{n_{sj}} \left[\boldsymbol{Z}_{sj,i_l} - \boldsymbol{Z}_{sj,m_{sj}+l} \right] \right) \right)^{-1} \tag{3}$$

For comparison of the approximate likelihood, we take a second order Taylor expansion of $\boldsymbol{f}(\widetilde{X}_{sj,i_l})$ at $\widetilde{X}_{sj,m_{sj}+l}$, then the approximate likelihood function is:

$$\begin{aligned}
\widetilde{L}_{sj} \approx & \Bigg(1 + \sum_{(i_1,\ldots,i_{n_{sj}})\in A'_{sj}} \exp\Big(\boldsymbol{\beta}_X^T \sum_{l=1}^{n_{sj}} \Big[\boldsymbol{f}(\widetilde{X}_{sj,m_{sj}+l}) + \boldsymbol{f'}(\widetilde{X}_{sj,m_{sj}+l})(\widetilde{X}_{sj,i_l} - \widetilde{X}_{sj,m_{sj}+l}) \\
& - \boldsymbol{f}(\widetilde{X}_{sj,m_{sj}+l}) + \text{remainder}\Big] + \boldsymbol{\beta}_{\boldsymbol{Z}}^T \sum_{l=1}^{n_{sj}} \left[\boldsymbol{Z}_{sj,i_l} - \boldsymbol{Z}_{sj,m_{sj}+l}\right]\Big)\Bigg)^{-1} \\
= & \Bigg(1 + \sum_{(i_1,\ldots,i_{n_{sj}})\in A'_{sj}} \exp\Big(\boldsymbol{\beta}_X^T \sum_{l=1}^{n_{sj}} \Big[\boldsymbol{f'}(\widetilde{X}_{sj,m_{sj}+l})(\widetilde{X}_{sj,i_l} - \widetilde{X}_{sj,m_{sj}+l}) + \text{remainder}\Big] \\
& + \boldsymbol{\beta}_{\boldsymbol{Z}}^T \sum_{l=1}^{n_{sj}} \left[\boldsymbol{Z}_{sj,i_l} - \boldsymbol{Z}_{sj,m_{sj}+l}\right]\Big)\Bigg)^{-1}
\end{aligned} \tag{4}$$

Note that, for the full calibration method, all observations have $\widetilde{X}_{sji} = E\left(X_{sji}|W_{sji}, Y_{sji} = 0\right) = a_{s,co} + b_{s,co}W_{sji}$. In the internalized calibration method, for the cases, $\widetilde{X}_{sj,m_{sj}+l} = a_{s,co} + b_{s,co}W_{sj,m_{sj}+l}$ for $l = 1, \ldots, n_{sj}$; while for $\widetilde{X}_{sj,i_l}$, part of them are replaced by X_{sj,i_l} if the observation was in the calibration subset; and for those that are not in the calibration subset, we have $\widetilde{X}_{sj,i_l} = a_{s,co} + b_{s,co}W_{sj,i_l}$.

Therefore, the approximate likelihood function under full calibration is:

$$\begin{aligned}
\widetilde{L}_{sj} \approx & \Bigg(1 + \sum_{(i_1,\ldots,i_{n_{sj}})\in A'_{sj}} \exp\Big(\boldsymbol{\beta}_X^T \sum_{l=1}^{n_{sj}} \Big[\boldsymbol{f'}(\widetilde{X}_{sj,m_{sj}+l})(\widetilde{X}_{sj,i_l} - \widetilde{X}_{sj,m_{sj}+l}) + \text{remainder}\Big] \\
& + \boldsymbol{\beta}_{\boldsymbol{Z}}^T \sum_{l=1}^{n_{sj}} \left[\boldsymbol{Z}_{sj,i_l} - \boldsymbol{Z}_{sj,m_{sj}+l}\right]\Big)\Bigg)^{-1} \\
= & \Bigg(1 + \sum_{(i_1,\ldots,i_{n_{sj}})\in A'_{sj}} \exp\Big(\boldsymbol{\beta}_X^T \sum_{l=1}^{n_{sj}} \Big[\boldsymbol{f'}(\widetilde{X}_{sj,m_{sj}+l})(a_{s,co} + b_{s,co}W_{sj,i_l} - a_{s,co} - b_{s,co}W_{sj,m_{sj}+l}) \\
& + \text{remainder}\Big] + \boldsymbol{\beta}_{\boldsymbol{Z}}^T \sum_{l=1}^{n_{sj}} \left[\boldsymbol{Z}_{sj,i_l} - \boldsymbol{Z}_{sj,m_{sj}+l}\right]\Big)\Bigg)^{-1} \\
= & \Bigg(1 + \sum_{(i_1,\ldots,i_{n_{sj}})\in A'_{sj}} \exp\Big(\boldsymbol{\beta}_X^T \sum_{l=1}^{n_{sj}} \Big[\boldsymbol{f'}(\widetilde{X}_{sj,m_{sj}+l})(b_{s,co}W_{sj,i_l} - b_{s,co}W_{sj,m_{sj}+l}) + \text{remainder}\Big] \\
& + \boldsymbol{\beta}_{\boldsymbol{Z}}^T \sum_{l=1}^{n_{sj}} \left[\boldsymbol{Z}_{sj,i_l} - \boldsymbol{Z}_{sj,m_{sj}+l}\right]\Big)\Bigg)^{-1}
\end{aligned} \tag{5}$$

The intercept of the calibration method cancels out in the approximated likelihood function, and therefore the bias from intercept $\widehat{a}_{s,co}$ does not affect the estimates of $\boldsymbol{\beta}_X$ under full calibration model.

For the internalized calibration, observations that are in the calibration subset retain their original value X_{sji}, and observations that are not in the calibration subset will have $\widetilde{X}_{sji} = a_{s,co} + b_{s,co} W_{sji}$. Suppose the calibration proportion is $C\%$. For each subset $(i_1, i_2, \ldots, i_{n_{sj}})$ of A'_{sj}, denote the proportion of observations that use reference laboratory measurements as $C'_{i_l}\%$, where $C'_{i_l}\% \leq C\%$. Without loss of generality, assuming observations that have reference laboratory measurements are listed before observations that do not have reference laboratory measurements in $(i_1, i_2, \ldots, i_{n_{sj}})$:

$$
\begin{aligned}
\widetilde{L}_{sj} \approx &\Bigg(1 + \sum_{(i_1,\ldots,i_{n_{sj}}) \in A'_{sj}} \exp\Big(\boldsymbol{\beta}_X^T \sum_{l=1}^{n_{sj} \times C'_{i_l}\%} \Big[\boldsymbol{f'}(\widetilde{X}_{sj,m_{sj}+l})(X_{sj,i_l} - \widetilde{X}_{sj,m_{sj}+l}) + \text{remainder}\Big] \\
&+ \sum_{l=n_{sj} \times C'_{i_l}\%+1}^{n_{sj}} \Big[\boldsymbol{f'}(\widetilde{X}_{sj,m_{sj}+l})(\widetilde{X}_{sj,i_l} - \widetilde{X}_{sj,m_{sj}+l}) + \text{remainder}\Big] + \boldsymbol{\beta}_{\boldsymbol{Z}}^T \sum_{l=1}^{n_{sj}} \big[\boldsymbol{Z}_{sj,i_l} - \boldsymbol{Z}_{sj,m_{sj}+l}\big]\Big)\Bigg)^{-1} \\
= &\Bigg(1 + \sum_{(i_1,\ldots,i_{n_{sj}}) \in A'_{sj}} \exp\Big(\boldsymbol{\beta}_X^T \sum_{l=1}^{n_{sj} \times C'_{i_l}\%} \Big[\boldsymbol{f'}(\widetilde{X}_{sj,m_{sj}+l})(X_{sj,i_l} - a_{s,co} - b_{s,co} W_{sj,m_{sj}+l}) + \text{remainder}\Big] \\
&+ \sum_{l=n_{sj} \times C'\%+1}^{n_{sj} \times C'_{i_l}\%} \Big[\boldsymbol{f'}(\widetilde{X}_{sj,m_{sj}+l})(b_{s,co} W_{sj,i_l} - b_{s,co} W_{sj,m_{sj}+l}) + \text{remainder}\Big] + \boldsymbol{\beta}_{\boldsymbol{Z}}^T \sum_{l=1}^{n_{sj}} \big[\boldsymbol{Z}_{sj,i_l} - \boldsymbol{Z}_{sj,m_{sj}+l}\big]\Big)\Bigg)^{-1}
\end{aligned} \tag{6}
$$

With the internalized calibration, the intercept $\widehat{a}_{s,co}$ and slope $\widehat{b}_{s,co}$ are both retained in the likelihood function, and therefore bias in $\widehat{a}_{s,co}$ would introduce additional bias in the estimates of $\boldsymbol{\beta}_X$ as compared to the full calibration method. Moreover, the bias will be more pronounced if the calibration proportion is large.

S5 Additional tables and figures

Table S1 Comparison of operating characteristics for β_X under the model P $(Y_{sji} = 1|X_{sji}) = \beta_{0sj} + \beta_{X_1} f_1(X_{sji}) + \beta_{X_2} f_2(X_{sji})$ for Internalized calibration(IN), Full calibration(FC) and Naive methods. Relative bias is computed by $\frac{\hat{\beta}-\beta}{\beta}$, and the reported value is the average over the 1000 simulations. Coverage rate is the proportion of simulations that yield a 95% confidence interval covering the true parameter. Standard deviation is the square root of the empirical variance of parameter estimates over all replicates; we report 10^3 times the standard deviation. The calibration proportion(denoted as Calib. size in the table) were set to be 5%, 15% and 30%. β_{X_2} is fixed at -0.16

		Relative bias of β_{X_1} (SD)			Coverage Rate of β_{X_1}			Relative bias of β_{X_2} (SD)			Coverage Rate of β_{X_2}		
Calib size	β_{X_1}	IN	FC	N	IN	FC	N	IN	FC	N	IN	FC	N
5%	$\log(1.25)$	-2.6%(3.309)	-4.0%(3.190)	-86.9%(0.120)	0.974	0.972	0.031	-2.9%(0.210)	-4.3%(0.206)	-91.7%(0.019)	0.951	0.950	0.000
	$\log(1.5)$	-1.8%(3.431)	-2.6%(3.245)	-50.9%(0.138)	0.965	0.962	0.021	-2.2%(0.265)	-3.6%(0.255)	-75.6%(0.021)	0.951	0.949	0.000
	$\log(1.75)$	-0.8%(4.578)	-1.3%(4.285)	-38.0%(0.177)	0.957	0.957	0.036	-0.9%(0.275)	-2.4%(0.268)	-61.9%(0.027)	0.934	0.931	0.013
	$\log(2)$	-1.3%(5.579)	-1.7%(5.218)	-31.2%(0.223)	0.966	0.966	0.048	-2.4%(0.322)	-3.9%(0.319)	-49.8%(0.035)	0.967	0.967	0.135
	$\log(2.25)$	-1.7%(5.672)	-2.1%(5.314)	-26.7%(0.279)	0.963	0.961	0.075	-3.0%(0.363)	-4.5%(0.369)	-38.1%(0.044)	0.957	0.952	0.378
	$\log(2.5)$	-1.3%(7.561)	-1.6%(7.191)	-23.1%(0.343)	0.967	0.959	0.122	-3.2%(0.480)	-4.8%(0.474)	-28.1%(0.054)	0.955	0.952	0.630
	$\log(2.75)$	-1.9%(7.568)	-2.2%(7.102)	-21.5%(0.375)	0.971	0.966	0.122	-4.8%(0.580)	-6.3%(0.574)	-21.5%(0.059)	0.953	0.958	0.788
15%	$\log(1.25)$	0.7%(1.213)	-3.5%(1.046)	-87.4%(0.116)	0.968	0.967	0.030	0.4%(0.106)	-3.9%(0.107)	-92.2%(0.019)	0.955	0.946	0.000
	$\log(1.5)$	-0.4%(1.387)	-2.6%(1.154)	-51.4%(0.137)	0.972	0.966	0.020	0.3%(0.113)	-3.9%(0.112)	-76.4%(0.021)	0.960	0.957	0.002
	$\log(1.75)$	0.0%(1.629)	-1.6%(1.406)	-38.1%(0.178)	0.967	0.960	0.039	1.1%(0.138)	-3.2%(0.140)	-62.0%(0.028)	0.956	0.956	0.015
	$\log(2)$	-0.8%(1.805)	-2.1%(1.550)	-31.1%(0.218)	0.954	0.947	0.043	-0.7%(0.166)	-5.1%(0.165)	-49.5%(0.033)	0.954	0.946	0.128
	$\log(2.25)$	-0.8%(2.097)	-1.8%(2.780)	-26.7%(0.276)	0.962	0.951	0.065	-0.1%(0.195)	-4.6%(0.194)	-38.8%(0.043)	0.957	0.945	0.368
	$\log(2.5)$	-0.1%(2.466)	-10.0%(2.141)	-23.2%(0.334)	0.961	0.955	0.116	1.3%(0.237)	-3.4%(0.236)	-28.4%(0.052)	0.955	0.955	0.628
	$\log(2.75)$	-0.9%(2.744)	-1.7%(2.423)	-21.1%(0.402)	0.953	0.949	0.167	-0.5%(0.257)	-5.3%(0.259)	-20.5%(0.063)	0.937	0.936	0.790
30%	$\log(1.25)$	5.2%(0.705)	-3.1%(0.514)	-88.1%(0.115)	0.967	0.956	0.022	5.5%(0.087)	-3.0%(0.086)	-92.4%(0.019)	0.950	0.951	0.000
	$\log(1.5)$	2.0%(0.884)	-2.4%(0.633)	-51.4%(0.137)	0.967	0.957	0.018	5.2%(0.094)	-3.4%(0.096)	-76.3%(0.022)	0.956	0.960	0.002
	$\log(1.75)$	1.4%(1.014)	-1.8%(0.754)	-37.7%(0.179)	0.954	0.943	0.038	5.4%(0.121)	-3.4%(0.121)	-61.1%(0.029)	0.944	0.939	0.021
	$\log(2)$	1.1%(1.154)	-1.4%(0.886)	-30.9%(0.223)	0.969	0.958	0.047	5.3%(0.136)	-3.6%(0.138)	-49.4%(0.034)	0.954	0.954	0.137
	$\log(2.25)$	-0.2%(1.522)	-2.3%(1.218)	-26.8%(0.277)	0.961	0.946	0.073	3.6%(0.174)	-5.3%(0.174)	-38.6%(0.043)	0.955	0.949	0.373
	$\log(2.5)$	0.1%(1.649)	-1.8%(1.312)	-23.4%(0.333)	0.960	0.956	0.110	4.6%(0.192)	-4.9%(0.193)	-28.9%(0.052)	0.942	0.941	0.617
	$\log(2.75)$	-1.9%(1.819)	-2.2%(1.529)	-21.5%(0.391)	0.971	0.966	0.122	-4.8%(0.242)	-6.3%(0.242)	-21.5%(0.061)	0.953	0.958	0.788

Table S2 Comparison of operating characteristics for β_X under the model P $(Y_{sji} = 1|X_{sji}) = \beta_{0sj} + \beta_{X_1} f_1(X_{sji}) + \beta_{X_2} f_2(X_{sji})$ for Internalized calibration(IN), Full calibration(FC) and Naive methods. Relative bias is computed by $\frac{\hat{\beta}-\beta}{\beta}$, and the reported value is the average over the 1000 simulations. Coverage rate is the proportion of simulations that yield a 95% confidence interval covering the true parameter. Standard deviation is the square root of the empirical variance of parameter estimates over all replicates; we report 10^3 times the standard deviation. The calibration proportion(denoted as Calib. size in the table) were set to be 5%, 15% and 30%. β_{X_1} is fixed at $\log(1.5) = 0.41$

		Relative bias of β_{X_1} (SD)			Coverage Rate of β_{X_1}			Relative bias of β_{X_2} (SD)			Coverage Rate of β_{X_2}		
Calib size	β_{X_2}	IN	FC	N	IN	FC	N	IN	FC	N	IN	FC	N
5%	-0.02	0.2%(3.593)	-0.5%(3.423)	-6.3%(0.182)	0.972	0.970	0.924	7.5%(0.278)	-4.4%(0.274)	194.0%(0.028)	0.962	0.961	0.644
	-0.06	-0.8%(3.794)	-1.5%(3.555)	-19.5%(0.169)	0.966	0.965	0.659	-2.2%(0.246)	-6.2%(0.240)	-12.1%(0.027)	0.950	0.948	0.925
	-0.10	-0.7%(3.604)	-1.5%(3.316)	-32.3%(0.153)	0.971	0.970	0.307	-1.6%(0.240)	-3.9%(0.235)	-52.9%(0.024)	0.963	0.961	0.376
	-0.14	-0.4%(3.374)	-1.1%(3.239)	-44.1%(0.144)	0.964	0.962	0.086	-1.0%(0.223)	-2.6%(0.225)	-69.8%(0.023)	0.949	0.946	0.013
	-0.18	-2.9%(3.636)	-3.6%(3.449)	-56.8%(0.135)	0.952	0.948	0.006	-3.6%(0.223)	-4.9%(0.228)	-79.5%(0.021)	0.943	0.937	0.000
15%	-0.02	2.4%(1.246)	0.1%(1.085)	-6.2%(0.189)	0.964	0.964	0.926	34.4%(0.134)	-3.4%(0.138)	191.3%(0.030)	0.949	0.956	0.634
	-0.06	1.4%(1.287)	-1.0%(1.150)	-19.6%(0.171)	0.966	0.957	0.650	8.0%(0.131)	-4.3%(0.132)	-12.9%(0.027)	0.955	0.957	0.928
	-0.10	1.5%(1.584)	-0.7%(1.379)	-32.2%(0.157)	0.962	0.960	0.297	3.4%(0.131)	-3.8%(0.128)	-53.9%(0.025)	0.948	0.948	0.343
	-0.14	-0.1%(1.472)	-2.3%(1.268)	-44.5%(0.141)	0.967	0.954	0.070	0.3%(0.114)	-4.7%(0.114)	-70.2%(0.023)	0.948	0.947	0.014
	-0.18	-0.1%(1.525)	-2.3%(1.295)	-56.5%(0.133)	0.965	0.960	0.004	0.6%(0.119)	-3.2%(0.117)	-79.4%(0.021)	0.957	0.953	0.000
30%	-0.02	4.5%(0.769)	0.0%(0.579)	-7.2%(0.190)	0.951	0.947	0.901	80.6%(0.108)	6.6%(0.109)	187.0%(0.030)	0.920	0.935	0.649
	-0.06	3.7%(0.895)	-0.8%(0.660)	-19.3%(0.171)	0.963	0.960	0.663	20.1%(0.109)	-4.1%(0.108)	-11.9%(0.027)	0.944	0.946	0.931
	-0.10	2.9%(0.864)	-1.6%(0.629)	-32.9%(0.152)	0.955	0.952	0.287	11.4%(0.098)	-2.9%(0.098)	-53.7%(0.024)	0.946	0.945	0.365
	-0.14	1.9%(0.862)	-2.5%(0.624)	-45.1%(0.141)	0.951	0.942	0.059	6.3%(0.096)	-3.5%(0.095)	-70.2%(0.021)	0.939	0.944	0.012
	-0.18	2.5%(0.902)	-1.9%(0.667)	-56.4%(0.139)	0.958	0.951	0.003	4.1%(0.099)	-3.4%(0.100)	-79.7%(0.022)	0.951	0.951	0.000

Table S3 Comparison of operating characteristics for β_X under the model P $= \beta(Y_{sji} = 1 | X_{sji}) = \beta_{X_1} f_1(X_{sji}) + \beta_{X_2} f_2(X_{sji})$ for Internalized calibration(IN), Full calibration(FC) and Naive methods with different $\frac{\sigma^2_{ws}}{\sigma^2_X}$. Relative bias is computed by $\frac{\hat{\beta}-\beta}{\beta}$, and the reported value is the average over the 1000 simulations. Coverage rate is the proportion of simulations that yield a 95% confidence interval trapping the true parameter. Standard deviation is the square root of the empirical variance of parameter estimates over all replicates; we report 10^3 times the standard deviation. The calibration proportion(denoted as Calib. size in the table) were set to be 5%, 15% and 30%. $\beta_{X_1} = 0.48, \beta_{X_2} = -0.16$

		Relative bias of β_{X_1} (SD)			Coverage Rate of β_{X_1}			Relative bias of β_{X_2} (SD)			Coverage Rate of β_{X_2}		
Calib size	$\frac{\sigma^2_{ws}}{\sigma^2_X}$	IN	FC	N	IN	FC	N	IN	FC	N	IN	FC	N
5%	0.75	-7.2%(23.705)	-11.0%(22.100)	-45.8%(0.174)	0.969	0.959	0.036	-9.3%(2.453)	-18.1%(2.325)	-71.5%(0.027)	0.970	0.954	0.007
	0.85	-5.2%(12.455)	-7.1%(11.877)	-45.1%(0.169)	0.97	0.961	0.034	-7.8%(0.843)	-12.3%(0.842)	-71.0%(0.026)	0.95	0.942	0.005
	0.90	-2.6%(7.949)	-3.9%(7.716)	-43.8%(0.156)	0.976	0.97	0.029	-4.0%(0.476)	-6.9%(0.497)	-69.2%(0.024)	0.963	0.957	0.002
	0.95	-1.5%(3.721)	-2.1%(3.543)	-44.0%(0.155)	0.97	0.967	0.023	-1.8%(0.212)	-3.1%(0.212)	-69.5%(0.024)	0.958	0.957	0.005
15%	0.75	0.9%(7.958)	-10.7%(6.451)	-46.6%(0.179)	0.983	0.947	0.033	9.0%(0.443)	-18.1%(0.467)	-72.7%(0.028)	0.961	0.916	0.011
	0.85	-0.5%(4.612)	-6.6%(3.923)	-45.5%(0.170)	0.978	0.58	0.022	3.6%(0.265)	-10.5%(0.263)	-71.0%(0.026)	0.957	0.947	0.007
	0.90	-0.4%(2.581)	-4.1%(2.136)	-44.0%(0.160)	0.971	0.954	0.031	0.9%(0.167)	-7.8%(0.167)	-70.0%(0.025)	0.953	0.949	0.002
	0.95	-0.4%(1.396)	-2.1%(1.178)	-43.6%(0.155)	0.97	0.962	0.028	-0.1%(0.119)	-4.1%(0.118)	-69.2%(0.025)	0.958	0.95	0.002
30%	0.75	12.4%(4.971)	-10.0%(3.205)	-45.6%(0.188)	0.948	0.941	0.04	34.8%(0.239)	-17.7%(0.253)	-71.7%(0.029)	0.816	0.916	0.006
	0.85	5.2%(2.380)	-6.7%(1.667)	-45.7%(0.162)	0.976	0.947	0.028	16.7%(0.160)	-10.9%(0.165)	-71.6%(0.026)	0.915	0.928	0.004
	0.90	4.1%(1.477)	-3.4%(0.990)	-43.8%(0.165)	0.957	0.955	0.031	11.6%(0.121)	-5.8%(0.124)	-69.0%(0.026)	0.933	0.941	0.005
	0.95	1.4%(0.859)	-2.2%(0.640)	-43.4%(0.160)	0.974	0.96	0.027	4.0%(0.104)	-4.2%(0.103)	-68.7%(0.025)	0.968	0.958	0.004

Table S4 Comparisonof operating characteristics for β_X under the model P $\left(Y_{sji}=1|X_{sji}\right)=\beta_{0sj}+\beta_{X_1}f_1(X_{sji})+\beta_{X_2}f_2(X_{sji})$ for Internalized calibration(IN), Full calibration(FC) and Naive methods. We set the nonlinear effect to be 0 (i.e. $\beta_{X_2}=0$). The point estimate of β_{X_2} is calculated, and the reported value is the average over the 1000 simulations. Coverage rate is the proportion of simulations that yield a 95% confidence interval covering 0. Standard deviation is the square root of the empirical variance of parameter estimates over all replicates. The calibration proportion(denoted as Calib. size in the table) were set to be 5%, 15% and 30%.

		Point estimate of β_{X_2} (SD)			Coverage Rate of β_{X_2}		
Calib size	β_{X_1}	IN	FC	N	IN	FC	N
5%	$\log(1.25)$	-0.001(0.043)	0.002(0.043)	-0.033(0.023)	0.947	0.949	0.690
	$\log(1.5)$	-0.003(0.045)	-0.001(0.045)	-0.060(0.025)	0.957	0.959	0.320
	$\log(1.75)$	-0.003(0.048)	-0.001(0.048)	-0.085(0.027)	0.960	0.961	0.099
15%	$\log(1.25)$	-0.003(0.044)	0.005(0.044)	-0.034(0.023)	0.954	0.947	0.685
	$\log(1.5)$	-0.008(0.045)	-0.001(0.045)	-0.061(0.025)	0.947	0.953	0.298
	$\log(1.75)$	-0.010(0.049)	-0.002(0.050)	-0.084(0.028)	0.934	0.936	0.129
30%	$\log(1.25)$	-0.013(0.044)	0.002(0.045)	-0.034(0.023)	0.932	0.933	0.685
	$\log(1.5)$	-0.014(0.043)	0.001(0.043)	-0.063(0.026)	0.959	0.966	0.281
	$\log(1.75)$	-0.016(0.048)	-0.001(0.048)	-0.084(0.028)	0.946	0.953	0.129

Table S5 Comparisonof operating characteristics for β_X under the model P $\left(Y_{sji}=1|X_{sji}\right)=\beta_{0sj}+\beta_{X_1}f_1(X_{sji})+\beta_{X_2}f_2(X_{sji})$ for Internalized calibration(IN), Full calibration(FC) and Naive methods. We set the nonlinear effect to be 0 (i.e. $\beta_{X_2}=0$). The point estimate of β_{X_2} is calculated, and the reported value is the average over the 1000 simulations. Coverage rate is the proportion of simulations that yield a 95% confidence interval covering 0. Standard deviation is the square root of the empirical variance of parameter estimates over all replicates. The calibration proportion(denoted as Calib. size in the table) were set to be 5%, 15% and 30%.

		Point estimate of β_{X_2} (SD)			Coverage Rate of β_{X_2}		
Calib size	β_{X_1}	IN	FC	N	IN	FC	N
5%	$-\log(1.25)$	-0.003(0.043)	-0.001(0.043)	0.033(0.023)	0.957	0.958	0.684
	$-\log(1.5)$	-0.003(0.045)	0.000(0.045)	0.060(0.025)	0.955	0.959	0.316
	$-\log(1.75)$	-0.004(0.047)	-0.002(0.047)	0.085(0.026)	0.965	0.962	0.081
15%	$-\log(1.25)$	-0.009(0.041)	-0.001(0.042)	0.032(0.023)	0.954	0.959	0.696
	$-\log(1.5)$	-0.010(0.046)	0.002(0.046)	0.060(0.024)	0.945	0.951	0.276
	$-\log(1.75)$	-0.008(0.050)	0.000(0.050)	0.082(0.024)	0.935	0.935	0.097
30%	$-\log(1.25)$	-0.016(0.044)	-0.002(0.045)	0.034(0.023)	0.933	0.940	0.667
	$-\log(1.5)$	-0.014(0.045)	0.000(0.045)	0.060(0.024)	0.932	0.945	0.298
	$-\log(1.75)$	-0.017(0.048)	-0.001(0.049)	0.082(0.027)	0.937	0.959	0.109

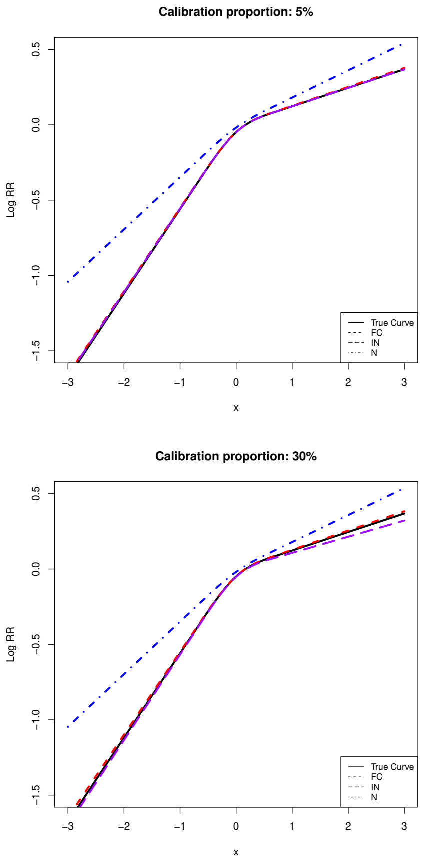

Figure S1 The curve reflecting the association of biomarker measurements on disease risk, where the x-axis is the biomarker value and y-axis is the log RR of the disease. The solid line is the true curve, and the dotted and dashed lines were estimated using internalized and full calibration method respectively, while the dashed-dotted line is estimated using naive method. The calibration proportion is 5%(left) and 30%(right), and the coefficients for the spline functions are set to be $\log(1.75) \approx 0.56$ and -0.16 respectively.
